# Cyclooxygenase-2 and its regulation in inflammation

**DOI:** 10.1155/S0962935196000452

**Published:** 1996-10

**Authors:** Y. S. Bakhle, R. M. Botting

**Affiliations:** 1 William Harvey Research Institute St Bartholomew's Hospital Medical College Charterhouse Square London EC1M 6BQ UK


Mediators of Inflammation, 5, 305–323 (1996)

### Cyclooxygenase-2 and its regulation in inflammation

Y. S. Bakhle[CA] and R. M. Botting

William Harvey Research Institute, St Bartholomew's Hospital Medical College, Charterhouse Square, London EC1M 6BQ, UK

[CA]Corresponding Author
Tel: (44) (0) 171 982 6119
Fax: (+44) (0) 171 251 1685

### Introduction

There is no doubt that the identification of an inducible isoform of cyclooxygenase (here referred to as cyclooxygenase-2 or COX-2) has brought about a renaissance in prostanoid biochemistry, pharmacology and therapeutics. This area is now as vigorous as it was 20 years ago when thromboxane $A_2$ ($TXA_2$) and prostacyclin ($PGI_2$) were discovered[1,2] and indeed is almost as active as that of nitric oxide (NO), with which it shares many features and correlations. One of the most important features of COX-2 is its close association with a variety of inflammatory mediators and its consequent description as the COX isoform involved in and responsible for many of the signs of inflammation. It is also generally accepted that COX-1 is the constitutive isoform involved in the physiological actions of prostaglandins (PGs) in the stomach and kidney, the inhibition of which leads to gastric ulceration and nephropathy as side effects of anti-inflammatory therapy with nonsteroidal anti-inflammatory drugs (NSAIDs).

The initial findings and many of the subsequent developments are based on the techniques of molecular biology[3,4] and are often expressed in terms unfamiliar to many researchers already established in inflammation. Our purpose in this review is to summarize the progress made so far in characterizing the regulation of COX-2, to evaluate its role in inflammation and, as a consequence, to assess the utility of the selective inhibitors of COX-2. In order to establish the appropriate context for the analysis of regulatory mechanisms, we shall first consider the molecular biochemistry of COX-2 and its possible place in physiology. We shall also refer to work on COX-1 where necessary.

### Molecular Biochemistry of COX-2

Although the early work on COX-2 utilized animal sources, information relating to the human form of this enzyme is steadily accumulating. Since the practical outcome of COX-2 research would be the more efficient alleviation of human inflammatory conditions, emphasis will be placed on results obtained with the human protein, along with data from animal sources wherever relevant. Here, for clarity and simplicity, the molecular biochemistry of COX-2 will be considered at three separate levels—its DNA, its RNA and the enzyme protein; further details of the molecular biology of COX-2 are available in two recent reviews.[3,4]

### DNA

The gene for COX-2 is located on chromosome 1 in both human and mouse cells.[5–8] The small size of the COX-2 gene (7.5–9 kbp[6,7,9]) is compatible with its inclusion in the group of inducible, immediate early genes, few of which are over 10 kbp in length.[10] It is relevant here to note that the human gene for $TXA_2$ synthase is larger (75 kbp[11]) and, like that for human $PGI_2$ synthase, is only weakly (two-fold) inducible.[12,13] The COX-2 gene has ten exons, one less than that for COX-1.[6,7] Overall, the descriptions from different groups, of human

© 1996 Rapid Science Publishers genomic DNA for COX-2[6,7,9] are in agreement and show many similarities between the human gene and the corresponding murine gene,[14] underlining the close relationship between species. The cDNA for human COX-2 was first derived from HUVEC cells.[15] More recently cDNA prepared from a human line of erythroleukaemia cells (HEL cells[7]) exhibited virtually an identical sequence with only two nucleotide differences.

In contrast to the similarity shown so far between species in the protein coding and 3′-flanking regions of COX-2 DNA, there are important species-related differences in the 5′-flanking region of the COX-2 gene, where the promoters and transcription factors bind. Whereas in the human gene there are putative binding sites for a variety of transcription factors including AP-2, SP-1, NF-IL6, NFkB and a cAMP responsive element (CRE) along with a TATA box and a TPA-response element in the first intron,[6,7,9,16,17] the corresponding region of the mouse gene appears to lack a CRE,[14] NFkB or NF-IL6 site,[7] although the others are present. The rat gene which has over 80% identity with the mouse gene in this region also lacks CRE, NFkB or AP-2 sites and a TATA box but includes a site for NF-IL6.[18,19] However more recent analysis of the mouse gene for COX-2 in an osteoblastic cell line has found an action of and sites for, NFkB; the same authors have also identified a NFkB binding site in the rat gene.[20]

### mRNA

Reflecting the similarity in cDNA for COX-2 across species, there is also considerable similarity in mRNA for COX-2, which at 4 kb is almost twice the size of that for COX-1 (2.8 kb) in all species so far examined.[21] Since the enzyme proteins (COX-1 and COX-2) are very similar in size, just over 600 amino acid residues, most of the difference in mRNA for COX-1 and COX-2 is taken up by the extensive 3′-untranslated region in COX-2 mRNA. This region includes several copies of the Shaw-Kamen 'instability' sequence, the actual number varying between species from 14 to 18 in animals.[22] In two examples of human mRNA, 17 and 22 copies were found.[7,9] These sequences are characteristic of rapidly degraded RNA[7,9] and have been found in the mRNA for other immediate early proteins.[23] However such sequences do not occur in the mRNA for COX-1 in any species.

Estimates of the half-life of COX-2 mRNA vary with the cells studied and with the stimuli used for induction of the protein. In an endothelium-derived cell line (ECV304[24]) with IL-1 as the inducing agent, COX-2 mRNA had a half-life of about 1 h. In the same system with transcription blocked, IL-1 was able to prolong the half-life of existing COX-2 mRNA to about 90 min, thus contributing also at a post-transcriptional stage to the overall induction of the enzyme. In an epithelial cell line (EGV6), COX-2 mRNA induced by the phorbol ester, TPA, had a half-life of 30 min.[25] It appears that the 'built-in' instability of the COX-2 mRNA is an essential component of the regulation of this protein and hence of its activity.

### Protein structure and function

The COX proteins are very similar, both between species and between isoforms, as they both carry out the same two separate catalytic functions, oxidation of arachidonate to $PGG_2$ and reduction of peroxide, specifically that of $PGG_2$ to $PGH_2$.[4,26,27] The differences in protein structure are small and chiefly outside what is considered to be the catalytically active site.[4,26]

The two isoforms are almost identical in size, COX-1 is about 602 residues whereas COX-2 comprises 604 residues. The major differences in sequence are at the N terminal where COX-2 has 17 less amino acids in the signal peptide and at the C-terminal where COX-2 has 18 more residues than COX-1.[4,26] The central parts of the proteins where the catalytic and substrate binding sites are located, are almost identical. The tyrosyl groups crucial for the oxidation and the histidines interacting with the haem group are all highly conserved as is the serine acetylated by aspirin.

### *(i) Substrate binding sites*

There are important functional differences between the isoforms which suggest that the active site in COX-2 is larger or has a looser fit than that in COX-1. This has been deduced from various mutations at the serine residue, which is acetylated by aspirin in either enzyme, Ser 530 in COX-1 or Ser 516 in COX-2.[28,29] (The different numbers for similarly placed residues in the two isoforms is due to the longer N terminal sequence in COX-1; the numbering for COX-1 is thus about 14 in advance of that for COX-2.) Mutation of serine to alanine in either isoform altered neither $K_m$ nor PG production but did confer protection against the irreversible inhibition caused by aspirin, since alanine cannot be acetylated.[29] However, mutation of serine to asparagine (isosteric with acetylated serine) has a strikingly differential effect; the COX-1 mutant lost cyclo-oxygenase activity whereas the COX-2 mutant retained full activity and an unchanged $K_m$. Substitution with a larger amino acid, glutamine, abolished cyclo-oxygenase activity in both isoforms.[28,29]

Another indication of the larger active site in COX-2 may be drawn from the effects of aspirin on catalytic activity. This compound irreversibly inhibits the production of PGs by COX-1 or COX-2 through the acetylation of Ser 530 or Ser 516. Nevertheless, acetylated COX-2 but not acetylated COX-1, still oxidized arachidonic acid (AA) to an alternative product, 15-HETE.[28–30] This finding would suggest that there is space for AA to bind to acetylated COX-2 close enough to the active site for oxidation to occur even though the orientation is not adequate for the full cyclo-oxygenase reaction to take place. Further support comes from the effect of another substitution of this serine in COX-2, with methionine; this leads to a 'pseudo-acetylated' form in that the mutant protein shows increased production of 15-HETE but with much reduced COX activity (5–20% of normal) and almost 200-fold increase in $K_m$.[28,29] Clearly with this substitution both the binding affinity and the binding mode were affected.

These deductions from biochemical findings were compatible with the model of COX-1 structure derived from X-ray crystallographic analysis.[31,32] The Ser 530 lies halfway along a tunnel leading up to the active site and it is relatively easy to imagine how the acetylation of Ser 530 would block access of substrates to the active site at the head of the tunnel. On the basis of the biochemical results for COX-2, one would assume that Ser 516 either lies further within the wall of the tunnel or that in COX-2 there is enough room for AA to 'squeeze past' the acetyl group and to bind close enough to the tyrosine-haem complex to allow oxidation to 15-HETE, but not to take up the configuration which leads to the cyclic endoperoxide ($PGG_2$).

Another possible factor is the residue on the opposite side of the tunnel, Ile523 in COX-1 which in COX-2 is substituted by Val509, one methylene group smaller than Ile. This location also provides the only difference between the active sites of the two isoforms. It is therefore possible that the extra methylene group of Ile 523 creates enough of a narrowing of the tunnel so that in combination with the acetyl group on Ser 530, access to the active site in COX-1 is essentially prevented. In COX-2, the presence of Val 509 would not only allow a wider range of fatty acid configurations to gain access as substrates in the normal enzyme but also provide less of a 'choke point' in the acetylated enzyme. Nevertheless, Val 509 was not important in binding of substrate as marked mutations at this site (Val to Lys or Glu) did not materially alter Km for AA of the COX-2 protein.[33]

An attempt to exploit the selectivity suggested by the larger substrate binding site in COX-2 had an unexpected outcome. It was argued that, as the acetylation of COX-2 still allows binding of AA to give 15-HETE, acylation with a larger acyl group should prevent any oxidation of AA by encroaching further into the binding area. In the event, the most potent analogue of aspirin was valeryl salicylic acid but it was a selective inhibitor of COX-1 with no inhibition of COX-2.[34] The explanation for this result is still to be put forward.

It is important to note that in the COX mutant proteins and in the acetylated native COX, the peroxidase activity catalysed by an active site on the other side of the haem group from that involved in the formation of $PGG_2$ remains unaffected, demonstrating quite clearly the separation of these two activities within the same protein molecule.[29,35,36]

*(ii) Inhibitor binding sites*

Because COX-1 and COX-2 are catalytically and structurally almost identical, it is likely that if selective inhibitors of COX-2 are to be found then these would not bind to any catalytically relevant site which would be the same for both isoforms, but to some other region possibly unique to COX-2. However access to substrate must still be denied in order to inhibit COX-2 activity. This line of reasoning would explain why most of the selective COX-2 inhibitors so far disclosed are not carboxylic acids (as are most COX-1 inhibitors) but interestingly contain a different common grouping, the sulphonamide or sulphone group.

Support for this suggestion for different binding sites for the two types of inhibitors comes from another mutant of COX-1 in which Arg 120 was replaced with Glu.[37,38] This positively charged residue (Arg 120) is located at the opening of the active site tunnel[31] and was assumed to be the binding site for the carboxylic acid group in the substrate fatty acids. It was also assumed to provide a binding site for the 'old', carboxylic acid, non-selective, COX inhibitors. These assumptions were fully confirmed by the characteristics of the (Arg 120–Glu) mutant COX-1, which showed a 100-fold higher Km for AA and a much reduced susceptibility to the carboxylic acid inhibitors including indomethacin, flurbiprofen and diclofenac all of which did not inhibit the mutant enzyme at concentrations between 200- and 8 000-fold higher than their $IC_{50}$ values for the wild-type enzyme.[37] However two COX-2 selective compounds, the sulphone DuP 697 and a sulphonamide analogue, were *more* potent as inhibitors of the mutant enzyme. Although this increased potency is probably due to the decreased binding of substrate AA (DuP 697 is a competitive reversible inhibitor of the wild-type COX-1 enzyme), there was clearly no *decrease* in the efficacy of the sulphonamide compounds and hence no loss of their binding to the mutant protein. In the other report,[38] the Arg 120–Glu mutant of ovine COX-1 showed no COX activity at all. Flurbiprofen binding was thus assessed with the Arg 120–Gln mutant which had 5% of the wild-type activity. This mutant did not show time-dependent inhibition and flurbiprofen's $IC_{50}$ value had increased from 5 μM (wild type) to 1 mM in the mutant. These results are probably adequate evidence for the importance of the Arg 120 residue in the binding of the 'old' non-selective inhibitors but the binding sites for the COX-2 selective inhibitors still remain to be determined, although two recent reports[33,39] provide some clues to its location.

In both, mutants of COX-2 have been generated with Val 509 being changed to Ile, as in COX-1, and, in the mutants, selective COX-2 inhibitors were much less potent and less capable of causing the time-dependent inhibition of COX-2, characteristic of wild-type COX-2. However in one report,[33] the COX-2 selective inhibitors (nimesulide, DuP697, NS398, SC58125) were still able to bind and inhibit presumably on a 'reversible' basis. The 'old' NSAIDs were unaffected by this mutation.[39] Here the Val/Ile substitution appears to be crucial in determining the activity and possibly selectivity of inhibitors of COX-2. The possible effects of this substitution have already been discussed above in terms of substrate access but it is less easy to visualize the crucial influence of a methylene group in the binding of strongly polar sulphone/sulphonamide compounds such as the selective COX-2 inhibitors. The crystal structure of human recombinant COX-2 was described at a recent meeting[40] and although the full report is not yet available some details relevant to this point have emerged. As expected the crystal structure of COX-2 is almost identical to that of COX-1. However for COX-2, two conformations appear to be possible for the binding of inhibitors,[40] one in which inhibitor binds to both Arg 120 and Tyr 355 (see Ref. 38) as polar sites (the closed conformer) and the other (open conformer) in which inhibitor binding excludes Arg 120. Since mutants of Arg 120 do not change binding of COX-2 selective inhibitors, the open conformer would appear to be the most likely form of COX-2 bound to a selective inhibitor. However these changes are at the mouth of the substrate tunnel and involve polar residues not the non-polar Val 509 which has such striking effects on inhibitor efficacy. It may thus be necessary to re-assess the role of another polar residue, Glu 524 (in COX-1). This negatively charged amino acid is close enough in the crystal[31,40] to form a salt bridge with the positively charged Arg 120. Although Glu 524 was not important for enzymic activity in COX-1,[38] it, along with Tyr 335 could provide polar binding sites in COX-2, alternative to Arg 120. This residue Glu 524/510 is also immediately adjacent to the Ile 523/Val 509 and it may be that the crucial effects of the Ile/Val substitution on inhibitor binding are actually to alter the configuration of the next residue, Glu 524/510. Clearly we need more information before the binding site for COX-2 selective inhibitors and the nature of the conformational change associated with their action are fully elucidated. (See Note Added in Proof).

The largest difference between the two proteins is the C-terminal extension (18 amino acids) and the lack of the 17 residues at the N-terminus in COX-2. Although the C-terminus in COX-1[31] and probably in COX-2, is also distant from the active site, it clearly exerts a considerable influence on the function of the enzyme as site-directed mutations at the extreme C-terminal of recombinant COX-1 had unexpectedly strong effects on activity.[41] Frustratingly, in COX-1 crystals, the X-ray analysis appears to extend reliably only to residue 586 of the 600 total residues in the ovine protein.[31] The three-dimensional structure of this C-terminal extension in COX-2 has also not been reported[40] although in the full report more information on this region may be presented. Mutants of COX-2 with alterations in this region will be invaluable in this analysis.

Another feature of inhibitor interaction with the COX proteins is the time-dependent irreversibility of some compounds (apart from aspirin). This varies between inhibitors and isoforms and is discussed further below but one aspect is relevant here. An inference from time-dependent and irreversible inhibition is that there may be chemical reaction between inhibitor and enzyme, for instance the formation of a Schiff base.[42] These interactions and subsequent conformational changes in protein structure[43] are different from acetylation by aspirin as all oxidation of AA is prevented, i.e. there is no formation of 15-HETE as in acetylated COX-2.[28–30] The selective, time-dependent inhibition of COX-2[42–45] would imply that conformational changes in COX-1 derive from interactions different from those that inactivate COX-2. It is important to note that neither reversible nor irreversible inhibitors affect the peroxidase activity of the proteins which continue to function normally in the presence of the inhibitors.[29,35,36] Another important consequence of the conformational rearrangement of the enzyme protein with inhibitor would be that the structure of COX-1 crystallized with inhibitor[31] could be different from that of enzyme crystallized with substrate, as already recognized.[46,47]

*(iii) Intracellular location and function*

The C-terminal region may also be especially important in securing the protein to the endoplasmic membranes. For both COX-1 and COX-2, the most commonly suggested location is in the membrane of the endoplasmic reticulum (ER) with an additional locus for COX-2 on the nuclear membrane.[48–50] Results from the crystallographic analysis suggested that the enzyme is bound to the ER by a sequence of short helical stretches of the molecule which probably only interact with one-half of the lipid bilayer, i.e. there is no transmembrane structure.[31] This description may now need to be modified. Ren *et al.*[51] using antibodies specific for particular sequences in COX-1 protein (including the C-terminus, the active site and glycosylation sites), together with selective lysis of cellular membranes have suggested that the C-terminus may cross the ER membrane to the cytoplasm, while agreeing with a luminal location for the rest of the molecule. Such a model would be compatible with the crystallographic analysis where the C-terminus beyond Arg 586 was not resolved and other results attributing importance to amino acid residues distant from the active site.[41,52] Although analogous mutations in COX-2 have not yet been assessed, these findings re-emphasize the importance of the structural analysis of the C-terminal region of the proteins.

The intracellular location of the COX protein is important because only COX-2 is found on the nuclear membrane where it would be ideally positioned to participate in mitogenesis, normal or neoplastic.

A possible mechanism for this participation is provided by recent findings, in three different systems, of the activation by $PGJ_2$ derivatives of the peroxisome proliferator activated receptor type gamma (PPARγ). The PPARs (α, β and γ) are members of the steroid hormone nuclear receptor family of proteins whose endogenous ligands are still undefined, although their activity as transcription factors is well established.[53] $PGD_2$, $PGJ_2$ and other related cyclopentenone PGs bind to PPARγ and cause the protein to act as a transcription factor for reporter genes[54–56] and also to stimulate the differentiation of fibroblasts into adipocytes.[54,55] These PGs may be the *endogenous* ligands for the PPARs, which might also explain the effects on cell growth already described for these prostanoids.[57–59] An intriguing finding is the stimulation of transcription of the haem oxygenase gene in rat cells by $PGJ_2$ derivatives[60,61] in relation to the possible role of haem oxygenase in the inflammatory response.[62]

The extent to which COX-2 accumulates in this location rather than in the ER could provide a control mechanism for this growth regulatory function, additional to any control of catalytic activity by inhibitors. The additional amino-acid residues in the C-terminus of COX-2 could be critical in the location of the enzyme in the nuclear membrane and spontaneous mutations in this region of the protein might affect the distribution between the ER and nuclear sites. More COX-2 in the nuclear membrane could, for instance, be associated with decreased apoptosis in epithelial cell lines.[63]

### Physiological and Pathophysiological Roles for COX-2

The accepted role for COX-2 is to provide PGs in a range of inflammatory and host defence conditions, which could be termed a 'pathological' role. Other functions such as involvement in mitogenesis and reproduction inferred from experimental results[3,18,19,57] could be considered as physiological or pathophysiological. These other roles have been supported by the results from 'knock-out' animals in which a gene coding for a particular protein is selectively inactivated. The logical purpose of knock-out strains is to expose deficiencies in the knock-out animal and from these defects to deduce the roles played by the missing protein. In the present context the relevant proteins are COX-1 and COX-2.

### Inflammation

Two separate reports[64,65] of COX-2 knock-out mouse strains (null mice) have appeared and in each a model of acute inflammation (oedema following AA applied to the ear) was used to test responses in the null mice. Both agreed that the null mice had normal ear oedema in response to AA and the interpretation of this result was that COX-1, still present, was able to generate the PGs required. Two other models of acute inflammation (PMA-ear oedema and carrageenin paw oedema) also gave normal results in null mice. The only model tested which failed in COX-2 null mice was one of LPS-induced hepatotoxicity which depends on induction of COX-2 in macrophages and/or Kupffer cells.[64] The implications of these experiments in mice lacking COX-2 are that where COX-1 is normally present (ear skin, paws) this isoform will substitute for the missing COX-2; where COX-1 is absent or at very low levels, as in macrophages, the inducing agent fails to generate the usual response.

### Reproduction and development

An essential role for COX-2 in embryonic development would be deduced from the severe morphological defects and consequent functional failures in the kidneys of COX-2 null mice,[64,65] which lead to their early death (some at about 8 weeks and most by 6 months). A similarly absolute requirement for COX-2 in female fertility must be deduced from the failure of null female mice to ovulate.[64] It is important to realize that these failures in null mice must represent a highly localized generation of COX products as the nature of the products from COX-2 is the same as those derived from COX-1 and COX-1 is still fully active in the COX-2 null mice.

A strong linkage between isoform and physiological function was further supported by the results from COX-1 knock-out mice.[66] Here the null mice are healthy, without developmental defects and with no ovulatory changes. However null pups carried by a null mother were mostly (> 90%) born dead; either a heterozygous mother or some heterozygous pups restored viability of the whole litter to normal. Clearly the COX-2 present in the COX-1 null mice cannot provide the PGs needed for foetal survival but COX-1 present in some littermates will ensure survival of all foetuses. Again the separate events in reproduction appear to have differing absolute requirements for COX isoforms.

### Implications of results from knock-out mice

The possibility of compensatory changes suggested for inflammation in COX-2 knock-out mice could also explain why in COX-1 null mice there was no gastric ulceration or NSAID-type nephropathy. Both these effects would have been predicted from the known effects of aspirin and other NSAIDs in normal animals. However in COX-1 null mice the compensation was not due to increased amounts of COX-2 activity as gastric tissue from the null mice synthesized less than 1% of the normal amount of PGs.[66] The most likely alternative compensation would be from NO; this mediator is, like $PGI_2$, a vasodilator and ulceration is associated with vasconstriction in the gastric microcirculation.[67] Like COX, both constitutive and induced NOS can be expressed in endothelium. One predictable consequence of this alternative pathway would be that NOS inhibitors would be ulcerogenic in the COX-1 null mice, whereas they are not noticeably so in normal animals.[67]

If the knock-out mice show that COX-1 and COX-2 have separate and important *physiological* roles in reproduction and COX-2, unlike COX-1, plays an essential part in foetal development, what do the knock-out mice tell us about their *pathological* importance in inflammation? One conclusion is that there is a clear need for COX-2 in certain forms of inflammation, perhaps all those related to the actions of LPS.[64] However the logical deduction from the other results is that COX-2 is not relevant to the inflammatory models used for many years to screen for anti-inflammatory compounds. On the basis of the knock-out results alone, several major pharmaceutical companies are wasting time, effort and money in searching for selective COX-2 inhibitors; these compounds will not decrease inflammation nor will they affect the incidence of NSAID-induced gastric ulcers as that effect is not connected with the presence or absence of COX-1 activity. In contrast to these logical deductions, there is a considerable body of empirical experimental evidence clearly demonstrating both the anti-inflammatory efficacy of selective COX-2 inhibitors and their decreased ulcerogenicity when compared with the more COX-1 selective inhibitors. This paradox is not without hope of resolution; there are still many relevant measurements to be made in the null strains—levels of COX-1 or COX-2 activity, of $PLA_2$ activity, the effect of selective inhibitors and many others—and when these results are gathered, another synthesis of the apparently opposing views may be possible.

### Regulation of COX-2 Activity

It is now clear that the activity of COX-2, as expressed by the synthesis of PGs, is normally controlled through the synthesis of the protein.

Several of the transcription factors effective on the COX-2 gene are known to be stimulated by inflammatory cytokines. Other cytokines and corticosteroids can alter the half-life of the inherently unstable mRNA, either increasing or decreasing translation into protein. For the protein itself, one option is to control the provision of substrate arachidonic acid (AA), although the most obvious regulator of activity would be a selective COX-1 or COX-2 enzyme inhibitor. All these possibilities, considered in more detail below, are summarized in Fig. 1.

### Regulation by inflammatory factors

A great variety of agents, mostly derived from inflammatory situations, have been used to induce COX-2 activity. The most frequently used inducing stimuli are IL-1 and lipopolysaccharide (LPS; used here as a synonym for bacterial endotoxin) and not as might have been expected the inflammatory stimuli often used for *in vivo* models, carrageenin[64,68–71] or zymosan.[72] These and other stimuli used with human cells are listed in Table 1.

### (i) Cytokines

Although IL-1 might be considered a model stimulus for the *local* induction of COX-2 in arthritis and LPS for the cardiovascular effects of the *systemic* induction of COX-2, these two stimuli are, *in vivo*, closely related as the effects of LPS are consequent on the production of TNFα and IL-1 from a range of cell types.

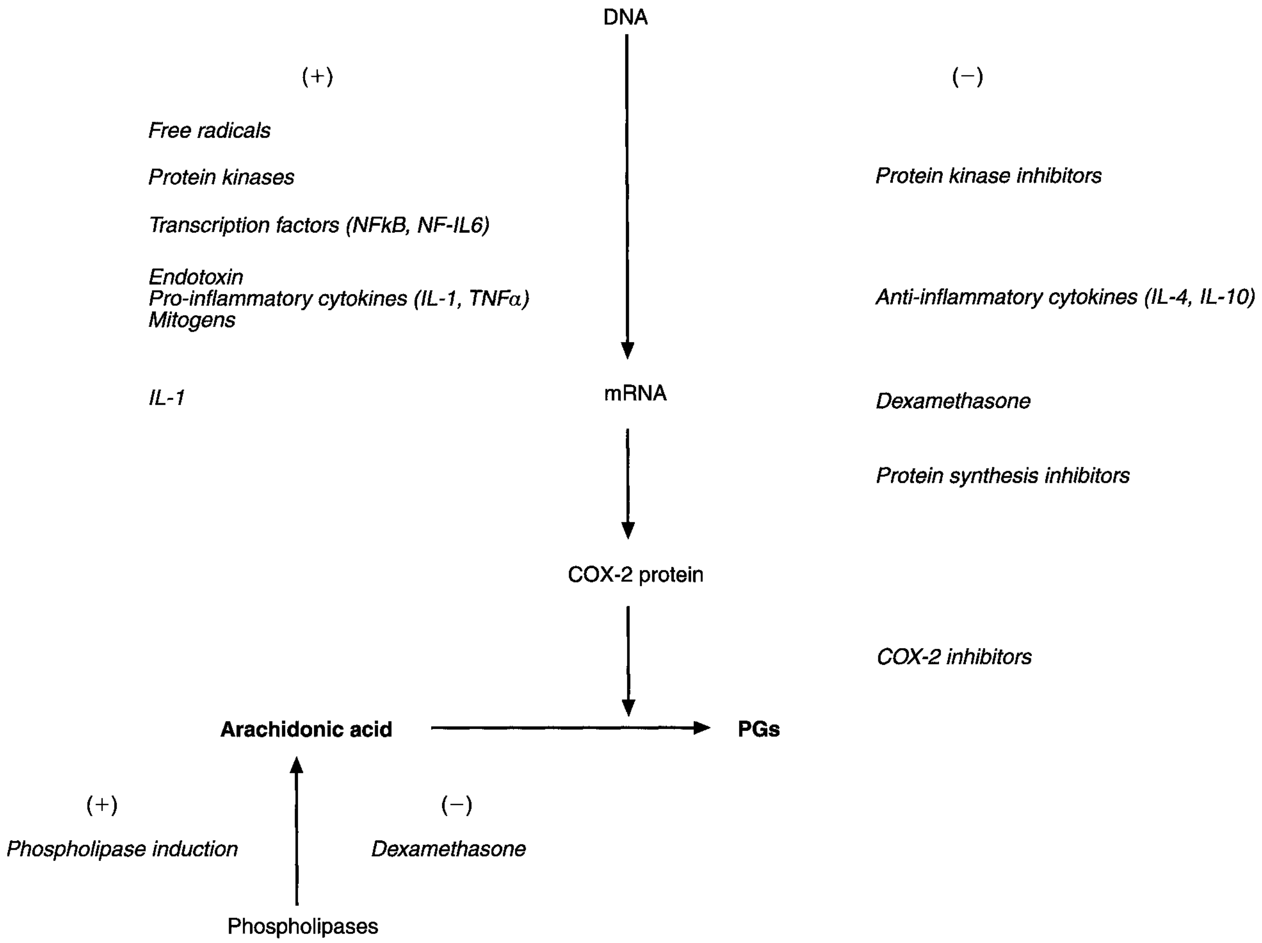

FIG. 1. Control of PG synthesis by regulation of COX-2 induction and action. This figure outlines the different stages at which modulation of COX-2 synthesis or function leads to alteration of the biological endpoint, production of PGs. Stimulatory factors (+) are shown on the left and inhibitory factors (−) on the right hand side. As discussed in the text, changes in the production of mRNA (transcription) by free radicals, protein kinases and cytokines are all finally mediated by changes in transcription factors. Because mRNA for COX-2 is inherently short-lived, alterations in its biological half-life can also significantly affect the amount of COX-2 protein synthesized. Protein synthesis inhibitors (cycloheximide) are included in this scheme for completeness but are not considered any further in the text as a means of regulating COX-2 levels. Note that factors affecting phospholipase induction or action could independently or co-operatively influence PG synthesis by altering the amounts of endogenous AA available to COX-2. The levels of PG production *in vivo* under physiological or pathological conditions will reflect the sum of changes in COX-2 and phospholipase activities. Intervention with COX-2 inhibitors allows exogenous control, overriding the endogenous mechanisms.

**Table 1.** Stimuli known to induce COX-2 mRNA, protein or activity in human cells

| Stimuli | Reference |
|---|---|
| LPS | 17, 76, 99, 108, 128, 130, 191–193 |
| TNF | 89, 193, 194 |
| IL-1 | 126, 127, 139, 143, 148, 157, 160, 171, 194–199 |
| EGF | 88, 89, 91, 200 |
| PDGF | 200, 201 |
| TGFα, β | 88, 89, 91 |
| Phorbol ester | 17, 148, 157, 202–204 |
| Pregnancy | 142, 203, 205, 206 |
| Parturition | 207–212 |

The references given are restricted to cells and tissues of human origin and to papers published in 1994, 1995 and early 1996. These are given as a guide to the range of systems (stimuli, cells, tissues or *in vivo*) used and not as a complete list of all the work on COX-2.

Differences between the effects of LPS and IL-1 in cultured cells will clearly depend on the ability of the cell line to release IL-1 in response to LPS; such differences are less likely *in vivo* or *ex vivo* where a wide range of cell types have been exposed to the inducing agent. However as at least two cytokines, IL-10 and IL-4, decrease induction (see below), their synthesis *in vivo* following IL-1 or LPS treatment could modify the final level of COX-2 activity attained.

In endothelial cells COX-2 is induced readily by LPS, in some cases through the release of TNFα, PDGF and other cytokines.[73–76] However the initial stage of this process, the binding of LPS to the cell membrane, is still unclear as endothelial cells do not express the particular LPS-binding membrane proteins (CD 14) that may be used as receptors on leukocytes,[77–79] a major cell type responding to LPS with induction of COX-2. There are some indications that soluble forms of CD 14 are involved in the mediation of responses to LPS in endothelial cells.[77,80]

Whereas most cytokines so far studied increase induction of COX-2, there are examples of *inhibition* by cytokines. Two interleukins, IL-10 and IL-4, already known to antagonize other effects of 'pro-inflammatory' cytokines,[81,82] decreased COX-2 levels in monocytes stimulated by LPS or Con A,[79,83,84] but in mast cells,[85,86] IL-10 potentiated, whereas IL-4 still inhibited, induction of COX-2 by c-kit ligand and IL-1. It is possible that this discrepancy in the effects of IL-10 is related to the cell types involved; more studies would be needed to define such a selectivity.

The TGF proteins, TGFα and TGFβ, present conflicting results for analysis. As might be expected from its mitogenic activity, TGFα was able to stimulate COX-2 production in epithelia[87] and to increase $PGE_2$ output in human amnion cells and in osteoblasts.[88,89] TGFα synergized with IL-1 or TNFα[90,91] to increase PG output, perhaps because TGFα can also induce IL-1 receptors.[91]

Although TGFβ potentiated the induction of COX-2 caused by phorbol ester in fibroblasts,[92,93] it had no effect when given alone. In macrophages,[94] the same cytokine inhibited the induction of COX-2 by LPS, more in keeping with its general anti-inflammatory profile. The apparent divergence of the effects of TGFβ may be more readily rationalized on the basis of cell types; for an overall anti-inflammatory and wound-healing effect, it would be reasonable to de-activate leukocytes and to stimulate fibroblasts at the same time. TGFβ also affects NOS induction in a range of cells[95] but here the majority of the results show a suppression of iNOS synthesis. The down-regulation of two major components of the inflammatory process would contribute importantly to the anti-inflammatory action of this cytokine.

*(ii) PGs*

The PGs themselves are inducing agents, as directly shown in osteocyte cultures[96–98] or by inference from the effects of COX inhibitors.[87,99,100] In osteoblastic cell (MC3T3) or calvarial cultures, $PGE_2$, $PGF_{2\alpha}$, $PGD_2$ and iloprost (a stable analogue of $PGI_2$) all induced COX-2 mRNA and protein[96–98] and indomethacin decreased COX-2 induction. Exogenous $PGE_2$ also stimulated COX-2 expression in mouse macrophages treated with LPS.[101] In these cells and in two other cell systems, rat epithelial cells stimulated with TGFα and phorbol ester and in human PMNs stimulated with LPS, COX inhibitors (indomethacin or sulindac sulphide) decreased COX-2 induction.[87,99] The positive feedback implied in these systems contrasts sharply with the negative feedback loops more usually found in inflammatory conditions where $PGE_2$ and other agonists that raise cAMP, actually decrease cytokine secretion from macrophages and lymphocytes.[102–104] Indeed the breaking of this loop with COX inhibitors (NSAIDs) in chronic inflammation is believed to increase cytokine production and subsequent degradation of joint cartilage while relieving pain and oedema; thus symptomatic benefit is undermined by a continuing or even accelerated disease process.[105,106]

*(iii) Free radicals and nitric oxide*

Another totally different class of compounds associated with inflammatory situations are the reactive oxygen intermediates or oxygen derived free radicals (ODFR), such as superoxide anion ($O_2^-$) and the hydroxyl radical ($OH^-$). A role for these species in the induction of COX-2 has also been proposed.[107,108] These highly reactive chemical species are present at sites of inflammation, leukocytes generate ODFR in the phagocytic process (the oxidative burst) and NO is another free radical, present during inflammation, synthesized by iNOS and capable of interacting with the ODFR to increase free radical actions. Thus, there are many opportunities for interactions between these radicals and COX-2. In rat mesangial cells following LPS, IL-1 or TNFα, radical scavengers (thioureas) or other antioxidants inhibited the usual increase in COX-2 mRNA, COX-2 protein and $PGE_2$ synthesis.[107] The effects were selective in that induction of other proteins (chemokines) by LPS or of COX-2 by other stimuli (phorbol esters, serum) were not similarly inhibited. At present, the actual mechanism of the interaction between the ODFR and the transcription of the COX-2 gene is not fully known, although ODFR are known to activate NFkB,[109,110] one of the transcription factors active on the COX-2 gene. However, in the rat the COX-2 gene may not exhibit a binding site for this transcription factor.[19,21]

Another free radical associated with inflammation is nitric oxide (NO) and its effects on COX activity remain difficult to summarize briefly. Both enzymes, COX and NO synthase (NOS), have inducible isoforms, which are induced in inflammatory situations by the same cytokines and both genes belong to the family of immediate early genes. Thus interaction between these two enzyme systems is highly likely; the difficulty arises from the outcome of that interaction. There was potentiation of PG output in the presence of NO,[111–115] apparently by direct interaction of NO with the haem in COX protein,[116] although this interpretation has been questioned.[117,118] The opposite effect, inhibition of PG output by NO, has also been observed in macrophages.[119] Action of PGs on NO output is less frequently encountered[120,121] although such effects might be deduced from the interactions of raised cAMP with NO output.[122–124] In one case a truly reciprocal relationship, with PGs inhibiting NO output and NO inhibiting PG output, by which a constant level of vasodilator tone may be maintained has been described in human saphenous vein.[125] This last example is unlikely to involve induced forms of either enzymes and is perhaps less relevant to the present discussion but it does illustrate the range of interactions possible between these enzymes. The most valid conclusion would seem to be that there is no generalization and that each situation with its particular combination of species, cell type and stimulus has to be evaluated individually.

*(iv) Contribution of phospholipases*

A potential source of confusion in the interpretation of experiments involving COX-2 induction is the simultaneous induction of $PLA_2$ activity, most frequently with IL-1 as inducing agent.[86,126–132] Increased $PLA_2$ activity implies increased provision of free AA which could be as easily available to COX-1 as to COX-2. Thus the final effect, increased synthesis of PGs, is not necessarily due solely to increased COX-2. One prediction from this co-induction is that the effectiveness of inhibitors of COX-2 could depend on the level of $PLA_2$ induced at the same time and that level could vary with the nature of the major inducing agent (IL-1, PAF or TNF) and with the cell type involved. The localization of the human gene for cytosolic $PLA_2$ to the same chromosomal region as the gene for COX-2 (1q25), raises the possibility of coordinate regulation of these enzymes.[133,134]

*(v) Effects of corticosteroids*

The major inhibitory mechanism affecting COX-2 induction, both in terms of experimental results and of pathophysiological relevance is the action of corticosteroids, most frequently demonstrated with dexamethasone (Dex). In many conditions, susceptibility of the production of PGs, enzyme protein or mRNA to inhibition by Dex is used as clear evidence for COX-2 induction. This simple conclusion is however clouded by other actions of Dex including the inhibition of the induction of $PLA_2$[132] and inhibition of $PLA_2$ activity itself via lipocortin.[135,136] For instance over 20 years ago it was shown that the output of PGs from freshly isolated lungs from untreated guinea-pigs, conditions in which COX-2 induction should be minimal, was decreased by infusions of Dex.[137] There is nevertheless clear evidence for decreased induction of both COX-2 protein and mRNA in the presence of Dex.[25,93,138–141]

There are also a few examples of corticosteroids increasing PG production. The mRNA for COX-2 but not that for COX-1 was increased 20-fold in human amnion cell cultures exposed to Dex for 16 h.[142] Interestingly, oestradiol and progesterone did not increase COX-2 mRNA but cortisol did. More typically, in human decidual tissue, Dex and progesterone were inhibitory.[143] As with other examples of corticosteroid inhibition of mRNA, the mechanism of action of Dex on COX-2 induction is poorly elucidated; either increased degradation of an already short-lived mRNA[8,144] or decreased transcription are possible contributors to the overall affect.

### Regulation via intracellular signalling

Tyrosine kinases play important roles in the intracellular signalling pathways for COX-2 induction in a range of cells; in endothelial cells, epithelial cells and macrophages this was demonstrated with tyrosine kinase inhibitors[73,74,145–148] and in mesangial cells by measurement of protein tyrosine phosphorylation.[72,149–151] The oncogene v-src encoding a tyrosine kinase is by itself enough to cause COX-2 induction in T cells.[144] Whereas the induction by EGF[25] probably utilized the receptor tyrosine kinase, the kinases used by other inducing agents have not yet been clearly identified. However the inhibition by tyrosine kinase inhibitors such as erbstatin and genistein of both COX-2 and iNOS induction[73,145,146,152] offers a new mode of anti-inflammatory action, which would have the advantage of not acting on constitutive enzyme activities. Such an effect may contribute to the anti-inflammatory properties of leflunomide[153] which was shown to inhibit the EGF-stimulated tyrosine kinase,[154] the Src tyrosine kinases[155] and the synthesis of $PGE_2$ induced by LPS in human leukocytes.[99]

Another signalling pathway, via protein kinase C (PKC), also appears to be involved but here the net effects of stimulating PKC activity are more variable. In most cases, increased PKC activity was associated with induction of COX-2[87,147,149,156–158] but in alveolar macrophages, *inhibition* of PKC with staurosporine caused COX-2 induction.[159] Another confusing factor is the well-established down-regulation of PKC on continued stimulation by phorbol esters. In the present context this was illustrated by the stimulation of COX-2 mRNA by 5HT (mediated via $5HT_2$ receptors and PKC activation) or by short exposure to phorbol ester. However *pre-incubation* with phorbol ester followed by 5-HT, abolished induction of COX-2 mRNA.[149]

These kinase pathways are not exclusive and in mesangial cells with 5HT as stimulus both PKC and tyrosine kinase mediate the induction of COX-2 mRNA.[149] In human skin fibroblasts after IL-1 stimulation, PKC appeared to be the major protein kinase with only minor contributions from PKA or tyrosine kinases.[160] However in ovarian tissue both PKA and PKC were activated during LH and GnRH stimulation of COX-2.[156] Other intracellular second messengers identified in osteoblasts include cAMP and PLC, stimulated by $PGE_2$ or iloprost (a stable analogue of $PGI_2$) and $PGF_{2\alpha}$ respectively.[96]

### Regulation by substrate

One of the continuing controversies in the analysis of COX action centres on the influence of different sources of substrate on activity. There is a clear difference between the utilization of endogenous and exogenous AA in that the supply of endogenous substrate is controlled by enzymes outside the COX cascade, chiefly by the balance between phospholipid hydrolysis and re- or trans-acylation.[161,162] The levels of endogenous substrate can be increased and PG output consequently stimulated in a number of cells and tissues (without induction of COX-2) by agents such as thrombin, histamine, bradykinin, PAF[163] or the crosslinking of IgE receptors.[164] This stimulation is characteristically of short duration, less than 30 min and there is no doubt that endogenous substrate is utilized by COX-1 to increase PG synthesis. The experiments in which COX-2 utilizes endogenous substrate are characteristically of longer duration (6–24 h) and entail incubation of cells with an inducing agent such as LPS, TPA or PDGF which is present throughout the incubation. The PGs accumulated over these longer periods are increased many-fold in the presence of the inducer molecule, relative to those in control incubations. Most of the inducing agents will increase phospholipase action as well as inducing COX-2 protein.[126,128–130,165] Again there is no doubt that COX-2 can utilize endogenous substrate to form PGs.

The problems appear with the use of exogenous substrate. Addition of exogenous AA (10–30 μM) to systems containing COX-1 leads to increased output of PGs. However in some preparations where both COX-1 and COX-2 are present, the induced enzyme appears to contribute no additional amount of PGs over that seen with COX-1 alone, leading to the proposition that COX-2 does not utilize exogenous AA.[92] Two comments are relevant. First, this 'inaccessibility' of exogenous AA is not a universal finding; in a variety of cells,[74,75,158,166,167] induction of COX-2 did lead to an increase in PG output from exogenous substrate. Second, the use of exogenous AA involves incubation for short times, typically 10 min, compared with 6 h incubations to show COX-2 induction.[92] Within 10 min over 100 ng $PGE_2$/ml was generated, whereas over 6 h there was accumulation of only about 20 ng/ml. An equally plausible explanation would be that although COX-2 was induced several-fold, the actual amounts of COX-2 protein were still low relative to the amount of COX-1 present. It is thus not possible to come to a definite conclusion on the selective accessibility of exogenous substrate to either isoform on the basis of these experiments.

A variation of this hypothesis was the conclusion drawn from work with only endogenous substrate in mast cells.[164] Here release of $PGD_2$ and COX-2 protein were induced by incubating the mast cells with a mixture of cytokines. Incubation in this medium for 5–10 h increased $PGD_2$ output many-fold to about 5–10 ng/ml. 'Acute' stimulation of $PGD_2$ biosynthesis by the crosslinking of IgE receptors in these cells was not increased until after 24 h incubation with the cytokine mixture, by which time the COX-2 protein had fallen again to near normal levels. There was certainly no increase in the 'acute' release of $PGD_2$ at the time of peak COX-2 induction. From this the authors concluded that the induced COX-2 did not have access to the increased AA released during IgE stimulated $PGD_2$ synthesis and that only COX-1 was utilized, even when COX-2 had been induced, to form PGs subsequent to IgE stimulation. A comparison of the times over which these experiments were performed shows that the IgE stimulation provided about 5 ng $PGD_2/10^6$ cells in 10 min whereas the cytokine stimulation took 10 h to provide 10 ng/$10^6$ cells. On this basis the induced COX-2 would provide about 0.2 ng in 10 min; this contribution would be rather difficult to detect against the total of 5 ng produced. The authors may be correct in postulating different coupling of stimuli to the isoforms but the experimental results are not an adequate test of their hypothesis. Indeed in a more recent paper from this group, the 'stimulus selective' linkage of COX-1 or COX-2 action has been replaced by a 'time selective' hypothesis.[168]

If distinct pools of substrate for COX-1 and COX-2 do exist they are more likely to be defined on a spatial basis than on a simple endogenous/exogenous substrate criterion. Indeed if COX-2 shares the general three-dimensional structure proposed for COX-1,[31] with the membrane anchors defining the entrance to the active site tunnel which guides AA cleaved from the adjacent membrane up to the oxidative site of the enzyme, it is not immediately obvious how one isoform could favour exogenous AA over the freshly hydrolysed product of the underlying ER membrane. However this model of COX action would assume a phospholipase in close proximity to the COX protein and a more likely basis of selectivity is in the phospholipase activated to supply endogenous AA. Although most emphasis has been placed on $PLA_2$ in this context, the action of either PLC or PLD can also give rise to free AA. Different ligands for cell membrane receptors will activate these phospholipases differentially. Furthermore, each phospholipase has its own substrate selectivities and the distribution of the phospholipids is not uniform throughout all membranes.[162] There is also evidence for a selective locus of COX-2 in the nuclear membrane, apart from the location in the ER demonstrable for both isoforms.[50] Thus a combination of which phospholipase is activated by which stimulus and which phospholipid is closest to a particular COX protein might *appear* to give a degree of selectivity between isoforms in terms of the substrate.

### Selective Inhibition of COX-2

One disadvantage of regulating COX-2 by interference with the processes of transcription, translation or intracellular signalling pathways is that, at present, selectivity of effect is low. This is well recognized for the corticosteroids which will prevent induction of many proteins apart from COX-2 and could be equally true for antagonists of or interference with, transcription factors such as NFkB or NF-IL6[169] or for the inhibitors of tyrosine kinase. Logically the most selective effect would be attained by inhibition of the enzyme protein and this consideration coupled with the effectiveness of COX inhibitors already known has led to an extensive search for new inhibitors with a selective action on COX-2. Particularly, such selective agents should be free of the most significant side effects associated with COX-1 inhibition, gastric ulcers and nephropathy.

### Assessment of selectivity

The initial stages of this search were concerned with assessment of the selectivity of the known NSAIDs or COX inhibitors and very soon established one major difficulty in the analysis, a marked variation in the selectivity ratio ($IC_{50}$ for COX-1:$IC_{50}$ for COX-2) for any given compound; a high ratio representing selectivity for COX-2 inhibition. For instance, ratios for indomethacin ranged from 20[170] to 0.1.[171] This variability is due to variation, at every level, in the experimental conditions of the assays. Different types of cell are used, derived from different species, as whole cells, homogenates, purified extracts or recombinant proteins expressed in bacterial, insect or animal cells. Further variation is introduced in the time of pre-incubation with inhibitor, the concentration of exogenous substrate or the use of endogenous substrate. The last three factors contribute significantly to the marked differences in selectivity. For instance with 10 min pre-incubation, indomethacin at 1.6 μM completely abolished COX-2 activity whereas COX-1 activity suffered only 50% inhibition. However, with no pre-incubation, indomethacin had an $IC_{50}$ of 13.5 μM for COX-1 whereas for COX-2 the $IC_{50}$ was over 1 000 μM.[172] Clearly two very different selectivity ratios would be calculated from $IC_{50}$ values obtained under these two experimental conditions. Time-dependent inhibition of COX-2 but not of COX-1 by NS 398 was the major reason for its selectivity with human recombinant enzymes.[43,44] A similar differential time-dependency was reported for CGP28238, another COX-2 selective inhibitor.[42] This feature is not the sole determinant of selectivity as several COX-1 inhibitors also show time-dependent inhibition of either isoform.[42,44,45]

Another source of variability with important practical consequences is the nature of the enzyme system used, i.e. in whole cells, homogenates or purified enzymes. Many groups have used human platelets as a source of COX-1 and a variety of cells (renal mesangial cells, macrophage cell lines, peripheral blood monocytes) stimulated with IL-1 or LPS to provide COX-2. The $IC_{50}$ values for indomethacin for whole cell preparations vary but are always lower than those reported for cell free preparations.[173] This is true also for selective inhibitors; the $IC_{50}$ for CGP28238 against COX-2 was 15 nM in whole cells but 750 nM even after prolonged preincubation with purified enzyme.[42] Similarly, increased potencies (lower $IC_{50}$) have been noted for aspirin, ibuprofen and even salicylate in whole cells compared with values obtained in broken cell or purified enzymes.[174] There are no obvious explanations for this phenomenon. Preferential concentration of the inhibitor in lipid of the ER membrane to give a locally higher concentration than in the bulk solution would not explain why inhibitory potency is lost in broken cells as crude homogenates after centrifugation would contain enzyme still attached to fragments of ER. One possibility is that in whole cells the concentration of free AA is kept low by restraining phospholipase activity (through low intracellular calcium, for instance) and increasing re- or trans-acylation into lipid.[161,162] Thus, the initial binding of the inhibitor to the enzyme in whole cells takes place with little or no competition from the substrate AA, allowing a maximal inhibitory effect. In a broken cell preparation the calcium concentration is much higher than the normal intracellular level, lipase activities and concentrations of AA could thus be higher, providing more competition for the binding of inhibitor. Even the time dependent irreversible inhibitors will show some reversal of inhibition when exposed to high AA concentrations.[42]

The value of a model system must lie in its ability to predict compounds with selective COX-2 inhibition *in vivo* and one test of that value is to assess the compounds already known to exhibit such activity alongside those NSAIDs with the worst side effect profiles.[175] Thus compounds such as NS398, SC58125 and CGP28238 must be clearly separated from NSAIDs such as piroxicam, azapropazone or ketoprofen. Most model systems achieve this separation and will probably be efficient screens for selective COX-2 inhibitors. However, it must be remembered that over the last 25 years, reliance on *in vitro* screening with COX purified from ram seminal vesicle (now known to be almost entirely COX-1) must have led to the rejection of many COX-2 selective inhibitors before they could be tested *in vivo* and a similar absolute reliance on a single *in vitro* screen for COX-2 could lead to similar mistakes.

### Progress in development of selective inhibitors

There were at least two examples of possible selective COX-2 inhibitors in the literature; both exhibited a good anti-inflammatory effect *in vivo* with little or no inhibition of the standard COX preparation from ram seminal vesicle together with low ulcerogenic activity. The first of these compounds, nimesulide, was patented over 20 years ago and has been available latterly in several European countries as an out-of-patent, non-prescription analgesic and anti-inflammatory agent.[176] The other, CGP28238, is closely related in structure (see Fig. 2) and was first reported in 1989.[177] A third close relative, NS398, was described many years later as a selective inhibitor of COX-2.[178]

Subsequent development has led to DuP 697,[179] SC 58125[180] and L-745,337,[181] with many other similar compounds less extensively studied.[42,182–185] The most striking feature of this new generation of NSAIDs is that none of them are carboxylic acids, like the 'old' NSAIDs, and all have the sulphone or sulphonamide grouping. The simplest (and perhaps simplistic) inference from this is that the selective COX-2 inhibitors bind to a site that is different from that used by the 'old' NSAIDs[37] and, as proposed earlier, would suggest a structure unique to the COX-2 protein which, at first sight, is most likely to be the 18-amino acid

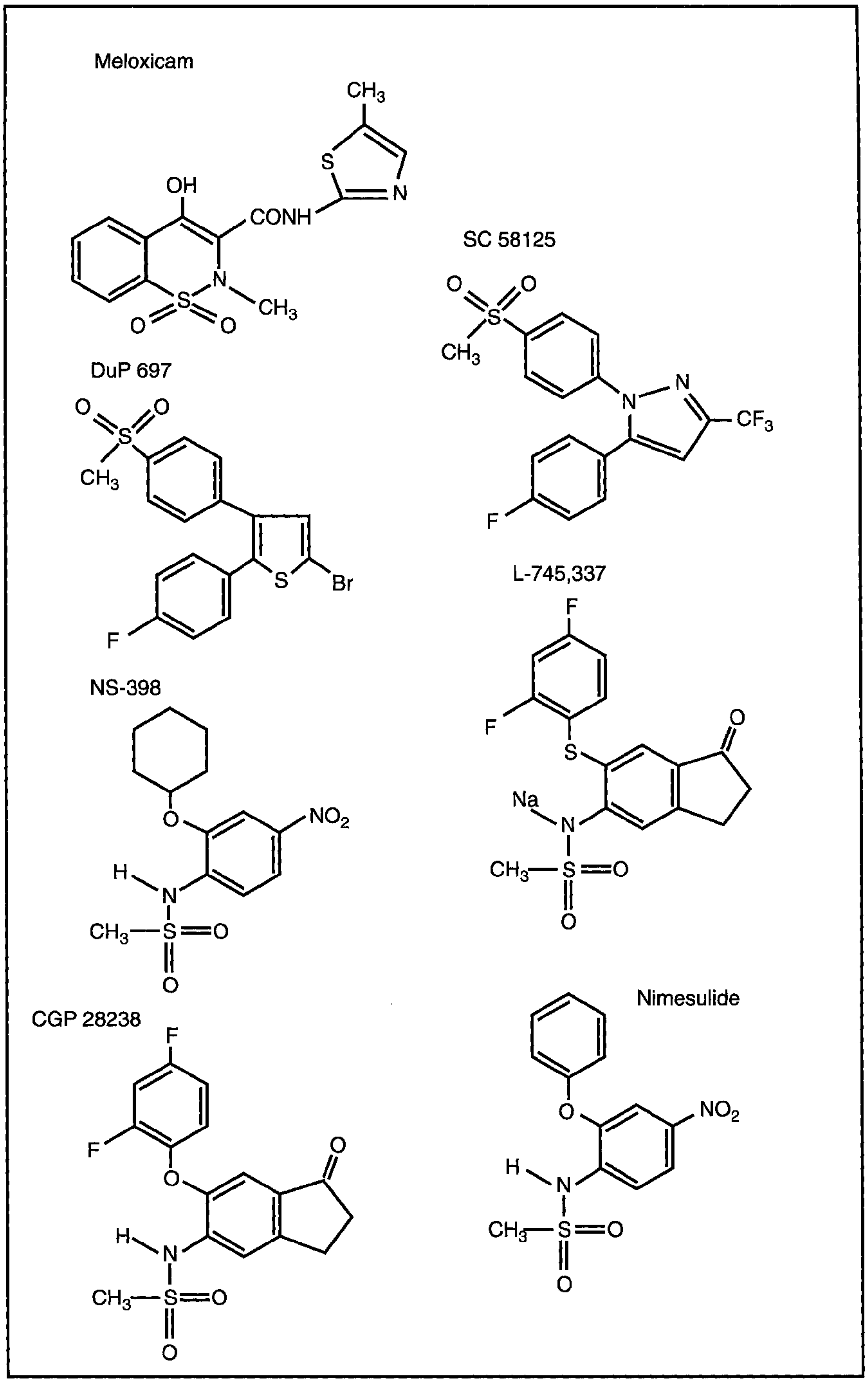

FIG. 2. Chemical structures of selective inhibitors of COX-2. All the compounds shown have exhibited inhibitory selectivity towards COX-2 in a range of systems (see Table 2). The sulphonamide grouping is present in all except meloxicam in which it forms part of a cyclic structure. None of these compounds has a carboxylic acid grouping characteristic of the 'older' NSAIDs.

insert at the C-terminus. It is important to note that neither the 'old' nor the new selective COX inhibitors affect the peroxidase activity of the proteins, implying that the binding of COX-2 inhibitors, like that of COX-1 inhibitors, does not disturb the three-dimensional structure of the protein on the 'other' side of the haem ring.[29,35,36]

In spite of all the reservations outlined above, the search for selective COX-2 inhibitors has been remarkably successful. To some extent this reflects the efficacy of the exemplar compound, nimesulide, but all the subsequent developments exhibit the predicted properties. They all have good selectivity *in vitro* with pure enzymes or in cell systems with ratios of $IC_{50}$

favouring COX-2 in all assays and some values are shown in Table 2. Furthermore they exert anti-inflammatory activity in a range of models, acute and chronic, as well as anti-pyretic and analgesic activities and at these doses there is little or no gastric ulceration. Clearly, it is possible to achieve selective inhibition of COX-2 and now it seems only a matter of refining the effective structures to combine the highest selectivity with the best pharmacokinetics and provide compounds for clinical evaluation.

One result of the selectivity of COX inhibition coupled with a better understanding of the conditions in which COX-2 can be induced is that it may now be reasonable to use COX-2 inhibitors in therapeutic areas such as endotoxin shock or asthma where the 'old' NSAIDs were ineffective. The greater potency against COX-2 together with lack of toxicity on stomach and kidney could allow a reduction in PG output via the induced enzyme while allowing the 'beneficial' output from constitutive COX-1. Another potential therapeutic area of considerable promise for COX-2 inhibitors is suggested by the negative correlation between colon cancer and NSAIDs;[186] recently aspirin was shown to reduce the risk of colorectal cancer by almost half.[187] The crucial observations were that the COX-2 isoform was present only in malignant tissue[188,189] and conferred resistance to apoptosis,[63,190] implying an important role for COX-2 in neoplastic growth.

The side effects or toxicity of COX-2 inhibitors are not easy to predict; certainly those of the 'old' NSAIDs should be absent, by definition. From the evidence of the knock-out mice,[64,65] the major toxicities will be on the reproductive system, on fertility and foetal development. Whereas decreased fertility, as long as it is reversible, may not be an unacceptable side effect, developmental defects, especially in the kidney, would most certainly prevent the use of COX-2 inhibitors during pregnancy and thus extensive testing for possible teratogenic effects would be required.

### Summary

Elucidation of the regulation of COX-2 provides an instructive example of the interaction between molecular biology and applied pharmacology. The basic science of the identification of the isoforms and the stimuli for induction was rapidly transformed into a new and powerful therapeutic concept, NSAIDs without the usual side effects. We now know a great deal about COX-2 from the gene to the crystal structure of the protein, its substrate sites and its intracellular location, much more than about many other enzymes of pharmacological importance. However in one significant aspect this encouraging utilization of molecular biology has failed; for all our knowledge, the design of selective inhibitors has not been based on a careful study of the structure of the protein and its interactions with substrate but, as in the past, on chemical variations of a molecular structure, found empirically to be effective. Moreover, deductions based on the knock-out mice would deny much of the equally empirical evidence correlating inflammation with COX activity.

Nevertheless it was molecular biology that disclosed the important place of COX-2 in reproduction, that raised new possibilities for

**Table 2.** Selective COX-2 inhibitors

| Compound | [a]$IC_{50}$ for COX-2 (nM) | [b]Ratio $IC_{50}$ COX-1:2 | [c]Anti-inflammatory doses (mg/kg) | | [d]Ulcerogenic potential *in vivo* (mg/kg) | References |
|---|---|---|---|---|---|---|
| | | | acute | chronic | | |
| Nimesulide | 13 | 13 | 3 | 0.2 | 100 | 213, 214 |
| CGP 28238 | 15 | 5000 | 2 | 0.05 | 30 | 171, 177 |
| NS 398 | 100 | 260 | 1 | 5 | > 1000 | 44, 215 |
| SC 58125 | 50 | > 2000 | 2 | – | > 600 | 180 |
| L 745337 | 23 | > 400 | 2 | – | > 30 | 181 |
| Meloxicam | 2 | 3 | 3 | 0.1 | 2.5 | 216, 217 |
| DuP 697 | 10 | 80 | 0.03 | 0.2 | > 400 | 45, 179 |
| *Indomethacin* | *6* | *0.03* | *1.5* | *0.1* | *8* | *215, 216* |
| *Piroxicam* | *175* | *0.03* | *2.7* | *0.6* | *1.1* | *216, 217* |

[a]These values were obtained *in vitro* with whole cells, purified native or recombinant enzymes.
[b]A high value here denotes high selectivity for inhibition of COX-2.
[c]The acute model was usually paw oedema, sometimes carrageenin pleurisy. The chronic model was usually adjuvant arthritis. The values shown are $ED_{50}$ or $ED_{30}$ doses in each model.
[d]These values represent the threshold dose for gastric damage or the highest dose at which damage was still absent.
Bearing in mind the diversity of test systems used, the important values in the table are not the absolute potencies of the compounds in any given test system but the ratios, firstly of $IC_{50}$ COX-1:COX-2 which gives some indication of selectivity *in vitro* (see text for further comment) and then the ratio of the $ED_{50}$ in chronic inflammation and the ulcerogenic dose, giving an estimate of *in vivo* selectivity. Note that the last two compounds in the table (shown in italics) are examples of the 'old, non-selective' NSAIDs. In fact these were quite selective for COX-1, as shown by the values for the $IC_{50}$ ratios.

the physiological role of COX-1 and that elucidated the correlation between COX-2 and neoplastic growth. In this last context, there is an intriguing possibility for which there is no direct evidence yet but which is entirely assessable with molecular biological techniques, namely that COX-2, like the products of other immediate-early genes, has effects on gene transcription and/or translation that do not entail the oxidation of AA to PGs. In all these roles and in the new ones to come, the analysis of regulatory mechanisms, physiological, pathophysiological and pharmacological, will remain central to scientific and clinical progress.

### Note Added in Proof

The X-ray crystal structure of human COX-2 desribed by M. Browner *et al.* is to appear in *Nature (Structural biology)* in November 1996. Two further descriptions, of murine COX-2 by R. G. Kurumbail *et al.* and of the human protein by B. M. McKeever *et al.* were given at a meeting in Vienna in September 1996 and have been submitted for publication. All three reports include discussions of the binding sites for selective inhibitors of COX-2.

### References

1. Hamberg M, Svensson J, Samuelsson B. Thromboxanes; a new group of biologically active compounds derived from prostaglandin endoperoxides. *Proc Natl Acad Sci USA* 1975; **71**: 345–349.
2. Moncada S, Gryglewski RJ, Bunting S, Vane JR. An enzyme isolated from arteries transforms prostaglandin endoperoxides to an unstable substance that inhibits platelet aggregation. *Nature* 1976; **263**: 663–665.
3. Herschman HR. Prostaglandin synthase 2. *Biochim Biophys Acta* 1996; **1299**: 125–140.
4. Otto JC, Smith WL. Prostaglandin endoperoxide synthases-1 and 2. *J Lipid Mediat Cell Signal* 1995; **12**: 139–156.
5. Tay A, Squire JA, Goldberg H, Skorecki K. Assignment of the human prostaglandin-endoperoxide synthase 2 (PTGS2) gene to 1q25 by fluorescence in situ hybridization. *Genomics* 1994; **23**: 718–719.
6. Tazawa R, Xu XM, Wu KK, Wang LH. Characterization of the genomic structure, chromosomal location and promoter of human prostaglandin H synthase-2 gene. *Biochem Biophys Res Commun* 1994; **203**: 190–199.
7. Kosaka T, Miyata A, Ihara H, Hara S, Sugimoto T, Takeda O, Takahashi E and Tanabe T. Characterization of the human gene (PTGS2) encoding prostaglandin-endoperoxide synthase 2. *Eur J Biochem* 1994; **221**: 889–897.
8. Xie W, Merrill JR, Bradshaw WS, Simmons DL. Structural determination and promoter analysis of the chicken mitogen-inducible prostaglandin G/H synthase gene and genetic mapping of the murine homolog. *Arch Biochem Biophys* 1993; **300**: 247–252.
9. Appleby SB, Ristimaki A, Neilson K, Narko K, Hla T. Structure of the human cyclo-oxygenase-2 gene. *Biochem J* 1994; **302**: 723–727.
10. Herschman HR. Primary response genes induced by growth factors and tumor promoters. *Annu Rev Biochem* 1991; **60**: 281–319.
11. Miyata A, Yokoyama C, Ihara H, Bando S, Takeda O, Takahashi E and Tanabe T. Characterization of the human gene (TBXAS1) encoding thromboxane synthase. *Eur J Biochem* 1994; **224**: 273–279.
12. Miyata A, Hara S, Yokoyama C, Inoue H, Ullrich V, Tanabe T. Molecular cloning and expression of human prostacyclin synthase. *Biochem Biophys Res Commun* 1994; **200**: 1728–1734.
13. Nanayama T, Hara S, Inoue H, Yokoyama C, Tanabe T. Regulation of two isozymes of prostaglandin endoperoxide synthase and thromboxane synthase in human monoblastoid cell line U937. *Prostaglandins* 1995; **49**: 371–382.
14. Fletcher BS, Kujubu DA, Perrin DM, Herschman HR. Structure of the mitogen-inducible TIS10 gene and demonstration that the TIS10-encoded protein is a functional prostaglandin G/H synthase. *J Biol Chem* 1992; **267**: 4338–4344.
15. Hla T, Neilson K. Human cyclooxygenase-2 cDNA. *Proc Natl Acad Sci USA* 1992; **89**: 7384–7388.
16. Inoue H, Nanayama T, Hara S, Yokoyama C, Tanabe T. The cyclic AMP response element plays an essential role in the expression of the human prostaglandin-endoperoxide synthase 2 gene in differentiated U937 monocytic cells. *FEBS Lett* 1994; **350**: 51–54.
17. Inoue H, Yokoyama C, Hara S, Tone Y, Tanabe T. Translational regulation of human prostaglandin-endoperoxide synthase-2 gene by lipopolysaccharide and phorbol ester in vascular endothelial cells. *J Biol Chem* 1995; **270**: 24965–24971.
18. Sirois J, Richards JS. Transcriptional regulation of the rat prostaglandin endoperoxide synthase 2 gene in granulosa cells. *J Biol Chem* 1993; **268**: 21931–21938.
19. Sirois J, Levy LO, Simmons DL, Richards JS. Characterization and hormonal regulation of the promoter of the rat prostaglandin endoperoxide synthase 2 gene in granulosa cells. *J Biol Chem* 1993; **268**: 12199–12206.
20. Yamamoto K, Arakawa T, Ueda N, Yamamoto S. Transcriptional roles of nuclear factor kB and nuclear factor-interleukin-6 in the tumor necrosis factor α-dependent induction of cyclooxygenase-2 in MC3T3-E1 cells. *J Biol Chem* 1995; **270**: 31315–31320.
21. Goppelt-Struebe M. Regulation of prostaglandin endoperoxide synthase (cyclooxygenase) isozyme expression. *Prostaglandins Leukotrienes Essential Fatty Acids* 1995; **52**: 213–222.
22. Feng L, Sun W, Xia Y, Tang WW, Chanmugam P, Soyoola E, Wilson CB and Hwang D. Cloning two isoforms of rat cyclooxygenase; differential regulation of their expression. *Arch Biochem Biophys* 1993; **307**: 361–368.
23. Shaw G, Kamen R. A conserved AU sequence from the 3′-untranslated region of GM-CSF mRNA mediates selective mRNA degradation. *Cell* 1986; **46**: 659–667.
24. Ristimaki A, Garfinkel S, Wessendorf J, Maciag T, Hla T. Induction of cyclooxygenase-2 by interleukin-1 alpha. Evidence for post-transcriptional regulation. *J Biol Chem* 1994; **269**: 11769–11775.
25. Hamasaki Y, Kitzler J, Hardman R, Nettesheim P, Eling TE. Phorbol ester and epidermal growth factor enhance the expression of two inducible prostaglandin H synthase genes in rat tracheal epithelial cells. *Arch Biochem Biophys* 1993; **304**: 226–234.
26. Smith WL, Marnett LJ. Prostaglandin endoperoxide synthases. *Metal Ions* 1994; **30**: 163–199.
27. Percival MD, Ouellet M, Vincent CJ, Yergey JA, Kennedy BP, O'Neill GP. Purification and characterization of recombinant human cyclooxygenase-2. *Arch Biochem Biophys* 1994; **315**: 111–118.
28. Mancini JA, O'Neill GP, Bayly C, Vickers PJ. Mutation of serine-516 in human prostaglandin G/H synthase-2 to methionine or aspirin acetylation of this residue stimulates 15-R-HETE synthesis. *FEBS Lett* 1994; **342**: 33–37.
29. Lecomte M, Laneuville O, Ji C, DeWitt DL, Smith WL. Acetylation of human prostaglandin endoperoxide synthase-2 (cyclooxygenase-2) by aspirin. *J Biol Chem* 1994; **269**: 13207–13215.
30. O'Neill GP, Mancini JA, Kargman S, Yergey J, Kwan MY, Falgueyret JP, Abramovitz M, Kennedy BP, Ouellet M, Cromlish W, Culp S, Evans JF, Ford-Hutchinson AW and Vickers PJ. Overexpression of human prostaglandin G/H synthase-1 and -2 by recombinant vaccinia virus: inhibition by nonsteroidal anti-inflammatory drugs and biosynthesis of 15-hydroxyeicosatetraenoic acid. *Mol Pharmacol* 1994; **45**: 245–254.
31. Picot D, Loll PJ, Garavito RM. The X-ray crystal structure of the membrane protein prostaglandin $H_2$ synthase-1. *Nature* 1994; **367**: 243–249.
32. Loll PJ, Picot D, Garavito RM. The structural basis of aspirin activity inferred from the crystal structure of inactivated prostaglandin $H_2$ synthase. *Nature Structural Biology* 1995; **2**: 637–643.
33. Guo Q, Wang L, Ruan K, Kulmacz RJ. Role of $Val^{509}$ in time-dependent inhibition of human prostaglandin H synthase-2 cyclooxygenase activity by isoform-selective agents. *J Biol Chem* 1996; **271**: 19134–19139.
34. Bhattacharyya DK, Lecomte M, Dunn J, Morgans DJ, Smith WL. Selective inhibition of prostaglandin endoperoxide synthase-1 (cyclooxygenase-1) by valerylsalicylic acid. *Arch Biochem Biophys* 1995; **317**: 19–24.
35. Tsai A, Hsi LC, Kulmacz RJ, Palmer G, Smith WL. Characterization of the tyrosyl radicals in ovine prostaglandin H synthase-1 by isotope replacement and site-directed mutagenesis. *J Biol Chem* 1994; **269**: 5085–5091.
36. Kulmacz RJ, Pendleton RB, Lands WE. Interaction between peroxidase and cyclooxygenase activities in prostaglandin-endoperoxide synthase. Interpretation of reaction kinetics. *J Biol Chem* 1994; **269**: 5527–5536.
37. Mancini JA, Riendeau D, Falgueyret JP, Vickers PJ, O'Neill GP. Arginine 120 of prostaglandin G/H synthase-1 is required for the inhibition by nonsteroidal anti-inflammatory drugs containing a carboxylic acid moiety. *J Biol Chem* 1995; **270**: 29372–29377.
38. Bhattacharyya DK, Lecomte M, Rieke CJ, Garavito RM, Smith WL.

Involvement of Arginine 120, Glutamate 524 and Tyrosine 355 in the binding of arachidonate and 2-phenylpropionic acid inhibitors to the cyclooxygenase active site of ovine prostaglandin endoperoxide H synthase-1. *J Biol Chem* 1996; **271**: 2179–2184.
39. Gierse JK, McDonald JJ, Hauser SD, Rangwala SH, Koboldt CM, Seibert K. A single amino acid difference between cyclooxygenase-1 (COX-1) and -2 (COX-2) reverses the selectivity of COX-2 specific inhibitors. *J Biol Chem* 1996; **271**: 15810–15814.
40. Browner MF. X-ray crystal structure of human cyclooxygenase-2. In: Bazan N, Botting JH, Vane JR, eds. *New Targets in Inflammation: Inhibitors of COX-2 or Adhesion Molecules*. London: Kluwer, 1996.
41. Ren Y, Loose-Mitchell DS, Kulmacz RJ. Prostaglandin H synthase-1: evaluation of C-terminus function. *Arch Biochem Biophys* 1995; **316**: 751–757.
42. Klein T, Nusing RM, Wiesenberg-Boettcher I, Ullrich V. Mechanistic studies on the selective inhibition of cyclooxygenase-2 by indanone derivatives. *Biochem Pharmacol* 1996; **51**: 285–290.
43. Copeland RA, Williams JM, Giannaras J, Nurnberg S, Covington M, Pinto D, Pick S and Trzaskos JM. Mechanism of selective inhibition of the inducible isoform of prostaglandin G/H synthase. *Proc Natl Acad Sci USA* 1994; **91**: 11202–11206.
44. Ouellet M, Percival MD. Effect of inhibitor time-dependency on selectivity towards cyclooxygenase isoforms. *Biochem J* 1995; **306**: 247–251.
45. Gierse JK, Hauser SD, Creely DP, Koboldt C, Rangwala SH, Isakson PC and Seibert K. Expression and selective inhibition of the constitutive and inducible forms of human cyclo-oxygenase. *Biochem J* 1995; **305**: 479–484.
46. Hsi LC, Hoganson CW, Babcock GT, Garavito RM, Smith WL. An examination of the source of the tyrosyl radical in ovine prostaglandin endoperoxide synthase-1. *Biochem Biophys Res Commun* 1995; **207**: 652–660.
47. Loll PJ, Garavito RM. The isoforms of cyclooxygenase: structure and function. *Expert Opinion on Investigational Drugs* 1994; **3**: 1171–1180.
48. Otto JC, Smith WL. The orientation of prostaglandin endoperoxide synthases-1 and -2 in the endoplasmic reticulum. *J Biol Chem* 1994; **269**: 19868–19875.
49. Regier MK, DeWitt DL, Schindler MS, Smith WL. Subcellular localization of prostaglandin endoperoxide synthase-2 in murine 3T3 cells. *Arch Biochem Biophys* 1993; **301**: 439–444.
50. Morita I, Schindler MS, Regier MK, Otto JC, Hori T, DeWitt DL and Smith WL. Different intracellular locations for prostaglandin endoperoxide H synthase-1 and -2. *J Biol Chem* 1995; **270**: 10902–10908.
51. Ren Y, Walker C, Loose-Mitchell DS, Deng J, Ruan K-H, Kulmacz RJ. Topology of prostaglandin H synthase-1 in the endoplasmic reticulum membrane. *Arch Biochem Biophys* 1995; **323**: 205–214.
52. Kennedy TA, Smith CJ, Marnett LJ. Investigation of the role of cysteines in catalysis by prostaglandin endoperoxide synthase. *J Biol Chem* 1994; **269**: 27357–27364.
53. Mangelsdorf DJ, Evans RM. The RXR heterodimers and orphan receptors. *Cell* 1995; **83**: 841–850.
54. Kliewer SA, Lenhard JM, Willson TM, Patel I, Morris DC, Lehmann JM. A prostaglandin $J_2$ metabolite binds peroxisome proliferator-activated receptor gamma and promotes adipocyte differentiation. *Cell* 1995; **83**: 813–819.
55. Forman BM, Tontonoz P, Chen J, Brun RP, Spiegelman BM, Evans RM. 15-Deoxy-delta-12,14-prostaglandin $J_2$ is a ligand for the adipocyte determination factor PPARgamma. *Cell* 1995; **83**: 803–812.
56. Yu K, Bayona W, Kallen CB, Harding HP, Ravera CP, McMahon G, Brown M and Lazar MA. Differential activation of peroxisome proliferator-activated receptors by eicosanoids. *J Biol Chem* 1995; **270**: 23975–23983.
57. Negishi M, Koizumi T, Ichikawa A. Biological actions of delta-12-prostaglandin $J_2$. *J Lipid Mediat Cell Signal* 1995; **12**: 443–448.
58. Fukushima M. Biological activities and mechanisms of action of $PGJ_2$ and related compounds; an update. *Prostaglandins Leukotrienes Essential Fatty Acids* 1992; **47**: 1–12.
59. Sasaki H, Fukushima M. Prostaglandins in the treatment of cancer. *Anticancer Drugs* 1994; **5**: 131–138.
60. Negishi M, Odani N, Koizumi T, Takahashi S, Ichikawa A. Involvement of protein kinase in delta-12-prostaglandin $J_2$-induced expression of rat heme oxygenase-1 gene. *FEBS Lett* 1995; **372**: 279–282.
61. Koizumi T, Odani N, Okuyama T, Ichikawa A, Negishi M. Identification of a cis-regulatory element for delta 12-prostaglandin $J_2$-induced expression of the rat heme oxygenase gene. *J Biol Chem* 1996; **270**: 21779–21784.
62. Willis D, Moore AR, Frederick R, Willoughby DA. Heme oxygenase: a novel target for the modulation of the inflammatory response. *Nature Medicine* 1996; **2**: 87–90.
63. Tsujii M, Dubois RN. Alterations in cellular adhesion and apoptosis in epithelial cells overexpressing prostaglandin endoperoxide synthase 2. *Cell* 1995; **83**: 493–501.
64. Dinchuk JE, Car BD, Focht RJ, Johnston JJ, Jaffee BD, Covington MB, Contel NR, Eng VM, Collins RJ, Czerniak PM, Gorry SA and Trzaskos JM. Renal abnormalities and an altered inflammatory response in mice lacking cyclooxygenase II. *Nature* 1995; **378**: 406–409.
65. Morham SG, Langenbach R, Loftin CD,Tiano HF, Vouloumanos N, Jenette JC, Mahler JF, Kluckman KD, Ledford A, Lee CA and Smithies O. Prostaglandin synthase 2 gene disruption causes severe renal pathology in the mouse. *Cell* 1995; **83**: 473–482.
66. Langenbach R, Morham SG, Tiano HF, Loftin CD, Ghanayem BI, Chulada PC, Mahler JF, Lee CA, Goulding EH, Kluckman KD, Kim HS and Smithies O. Prostaglandin synthase 1 gene disruption in mice reduces arachidonic acid-induced inflammation and indomethacin-induced gastric ulceration. *Cell* 1995; **83**: 483–492.
67. Whittle BJR. Neuronal and endothelium-derived mediators in the modulation of the gastric microcirculation: integrity in the balance. *Br J Pharmacol* 1993; **110**: 3–17.
68. Tomlinson A, Appleton I, Moore AR, Gilroy DW, Willis D, Mitchell JA and Willoughby DA. Cyclo-oxygenase and nitric oxide synthase isoforms in rat carrageenin-induced pleurisy. *Br J Pharmacol* 1994; **113**: 693–698.
69. Salvemini D, Manning PT, Zweifel BS, Seibert K, Connor J, Currie MG, Needleman P and Masferrer JL. Dual inhibition of nitric oxide and prostaglandin production contributes to the antiinflammatory properties of nitric oxide synthase inhibitors. *J Clin Invest* 1995; **96**: 301–308.
70. Katori M, Harada Y, Hatanaka K, Majima M, Kawamura M, Ohno T, Aizawa A and Yamamoto S. Induction of prostaglandin H synthase-2 in rat carrageenin-induced pleurisy and effect of a selective COX-2 inhibitor. *Adv Prostaglandin Thromboxane Leukotrienes Res* 1995; **23**: 345–347.
71. Harada Y, Hatanaka K, Kawamura M, Saito M, Ogino M, Majima M, Ohno T, Ogino K, Yamamoto K, Taketani Y, Yamamoto S and Katori M. Role of prostaglandin H synthase-2 in prostaglandin $E_2$ formation in rat carrageenin-induced pleurisy. *Prostaglandins* 1996; **51**: 19–33.
72. Glaser KB, Sung A, Bauer J, Weichmann BA. Regulation of eicosanoid biosynthesis in the macrophage. Involvement of protein tyrosine phosphorylation and modulation by selective protein tyrosine kinase inhibitors. *Biochem Pharmacol* 1993; **45**: 711–721.
73. Akarasereenont P, Bakhle YS, Thiemermann C, Vane JR. Cytokine-mediated induction of cyclo-oxygenase-2 by activation of tyrosine kinase in bovine endothelial cells stimulated by bacterial lipopolysaccharide. *Br J Pharmacol* 1995; **115**: 401–408.
74. Akarasereenont P, Mitchell JA, Appleton I, Thiemermann C, Vane JR. Involvement of tyrosine kinase in the induction of cyclo-oxygenase and nitric oxide synthase by endotoxin in cultured cells. *Br J Pharmacol* 1994; **113**: 1522–1528.
75. Akarasereenont P, Mitchell JA, Bakhle YS, Thiemermann C, Vane JR. Comparison of the induction of cyclooxygenase and nitric oxide synthase by endotoxin in endothelial cells and macrophages. *Eur J Pharmacol* 1995; **273**: 121–128.
76. Flynn JT, Hoff H. Lipopolysaccharide induces time-dependent increases in prostaglandin H synthase-2- and cytosolic phospholipase A2 mRNA in cultured human microvessel-derived endothelial cells. *Shock* 1995; **4**: 443–440.
77. Pugin J, Schurer-Maly CC, Leturcq D, Moriarty A, Ulevitch RJ, Tobias PS. Lipopolysaccharide activation of human endothelial and epithelial cells is mediated by lipopolysaccharide-binding protein and soluble CD14. *Proc Natl Acad Sci USA* 1993; **90**: 2744–2748.
78. Goldblum SE, Brann TW, Ding X, Pugin J, Tobias PS. Lipopolysaccharide (LPS)-binding protein and soluble CD14 function as accessory molecules for LPS-induced changes in endothelial barrier function *in vitro*. *J Clin Invest* 1994; **93**: 692–702.
79. Yano T, Hopkins HA, Hempel SL, Monick MM, Hunninghake GW. Interleukin-4 inhibits lipopolysaccharide-induced expression of prostaglandin H synthase-2 in human alveolar macrophages. *J Cell Physiol* 1995; **165**: 77–82.
80. Ulevitch RJ, Tobias PS. Recognition of endotoxin by cells leading to transmembrane signalling. *Curr Opin Immunol* 1994; **6**: 125–130.
81. Moore KW, O'Garra A, de Waal Malefyt R, Vieira P, Mossman TR. Interleukin-10. *Annu Rev Immunol* 1993; **11**: 165–190.
82. te Velde AA, Huijbens RJF, Heije K, de Vries JE, Figdor CG. Interleukin-4 (IL-4) inhibits secretion of IL-1α, tumor necrosis factor-α and IL-6 by human monocytes. *Blood* 1990; **76**: 1392–1397.
83. Mertz PM, DeWitt DL, Stetler-Stevenson WG, Wahl LM. Interleukin 10 suppression of monocyte prostaglandin H synthase-2. Mechanism of inhibition of prostaglandin-dependent matrix metalloproteinase production. *J Biol Chem* 1994; **269**: 21322–21329.
84. Niiro H, Otsuka T, Tanabe T, Hara S, Kuga S, Nemoto Y, Tanaka Y, Nakashima H, Kitajima S, Abe M and *et al.* Inhibition by interleukin-10 of inducible cyclooxygenase expression in lipopolysaccharide-stimulated monocytes: its underlying mechanism in comparison with interleukin-4. *Blood* 1995; **85**: 3736–3745.
85. Murakami M, Penrose JF, Urade Y, Austen KF, Arm JP. Interleukin-4 suppresses c-kit ligand-induced expression of cytosolic phospholipase A2 and prostaglandin endoperoxide synthase 2 and their roles in separate pathways of eicosanoid synthesis in mouse bone marrow-derived mast cells. *Proc Natl Acad Sci USA* 1995; **92**: 6107–6111.
86. Murakami M, Austen KF, Arm JP. The immediate phase of c-kit ligand stimulation of mouse bone marrow derived mast cells elicits rapid leukotriene C4 generation through post-translational activation of cytosolic phospholipase A2 and 5-lipoxygenase. *J Exp Med* 1995; **182**: 197–206.
87. Dubois RN, Awad J, Morrow J, Roberts LJ, Bishop PR. Regulation of eicosanoid production and mitogenesis in rat intestinal epithelial cells by transforming growth factor-alpha and phorbol ester. *J Clin Invest* 1994; **93**: 493–498.
88. Tahara M, Tasaka K, Masumoto N, Adachi K, Adachi H, Ikebuchi Y, Kurachi H and Miyake A. Expression of messenger ribonucleic acid for epidermal growth factor (EGF), transforming growth factor (TGF-alpha) and EGF receptor in human amnion cells; possible role of TGFalpha in prostaglandin E2 synthesis and cell proliferation. *J Clin Endocrinol Metab* 1995; **80**: 138–145.
89. Bry K, Hallman M, Lappalainen U. Cytokines released by granulocytes and mononuclear cells stimulate amnion cell prostaglandin $E_2$ production. *Prostaglandins* 1994; **48**: 389–399.
90. Harrison JR, Lorenzo JA, Kawaguchi H, Raisz LG, Pilbeam C. Stimulation of prostaglandin E2 production by interleukin-1 alpha and transforming growth factor alpha in osteoblastic MC3T3-E1 cells. *J Bone Miner Res* 1994; **9**: 817–823.
91. Bry K. Epidermal growth factor and transforming growth factor-alpha enhance the interleukin-1 and tumor necrosis factor stimulated prostaglandin $E_2$ production and the interleukin-1 specific binding on amnion cells. *Prostaglandins Leukotrienes Essential Fatty Acids* 1993; **49**: 923–928.
92. Reddy ST, Herschman HR. Ligand-induced prostaglandin synthesis requires expression of the TIS10/PGS-2 prostaglandin synthase gene in murine fibroblasts and macrophages. *J Biol Chem* 1994; **269**: 15473–15480.
93. Gilbert RS, Reddy ST, Kujubu DA, Xie W, Luner S, Herschman HR. Transforming growth factor beta 1 augments mitogen-induced prostaglandin synthesis and expression of the TIS10/prostaglandin synthase 2 gene both in Swiss 3T3 cells and in murine embryo fibroblasts. *J Cell Physiol* 1994; **159**: 67–75.
94. Reddy ST, Gilbert RS, Xie W, Luner S, Herschman HR. TGF-beta 1 inhibits both endotoxin-induced prostaglandin synthesis and expression of the TIS10/prostaglandin synthase 2 gene in murine macrophages. *J Leuk Biol* 1994; **55**: 192–200.
95. Vodovotz Y, Bogdan C. Control of nitric oxide synthase expression by transforming growth factor-beta; implications for homeostasis. *Prog Growth Factor Res* 1994; **5**: 341–351.
96. Takahashi Y, Taketani Y, Endo T, Yamamoto S, Kumegawa M. Studies on the induction of cyclooxygenase isozymes by various prostaglandins in mouse osteoblastic cell line with reference to signal transduction pathways. *Biochim Biophys Acta* 1994; **1212**: 217–224.
97. Pilbeam CC, Raisz LG, Voznesensky O, Alander CB, Delman BN and Kawaguchi H. Autoregulation of inducible prostaglandin G/H synthase in osteoblastic cells by prostaglandins. *J Bone Miner Res* 1995; **10**: 406–414.
98. Kawaguchi H, Raisz LG, Voznesensky OS, Alander CB, Hakeda Y, Pilbeam CC. Regulation of the two prostaglandin G/H synthases by parathyroid hormone, interleukin-1, cortisol, and prostaglandin $E_2$ in cultured neonatal mouse calvariae. *Endocrinology* 1994; **135**: 1157–1164.
99. Weithmann KU, Jeske S, Schlotte V. Effect of leflunomide on constitutive and inducible pathways of cellular eicosanoid generation. *Agents Actions* 1994; **41**: 164–170.
100. Kawaguchi H, Pilbeam CC, Gronowicz G, Abreu C, Fletcher BS, Herschman HR, Raisz LG and Hurley MM. Transcriptional induction of prostaglandin G/H synthase-2 by basic fibroblast growth factor. *J Clin Invest* 1995; **96**: 923–930.
101. Tordjman C, Coge F, Andre N, Rique H, Spedding M, Bonnet J. Characterisation of cyclooxygenase 1 and 2 expression in mouse resident peritoneal macrophages *in vitro*; interactions of non steroidal anti-inflammatory drugs with COX-2. *Biochim Biophys Acta* 1995; **1256**: 249–256.
102. Mohr C, Davis GS, Graebner C, Hemenway DR, Gemsa D. Enhanced release of prostaglandin $E_2$ from macrophages of rats with silicosis. *Am J Respir Cell Mol Biol* 1992; **6**: 390–396.
103. Renz H, Gong J-H, Schmidt A, Nain M, Gemsa D. Release of tumor necrosis factor-α from macrophages. Enhancement and suppression are dose-dependently regulated by prostaglandin $E_2$ and cyclic nucleotides. *J Immunol* 1995; **141**: 2388–2393.
104. Haynes DR, Whitehouse MW, Vernon-Roberts B. The prostaglandin $E_1$ analogue, Misoprostol, regulates inflammatory cytokines and immune functions *in vitro* like the natural prostaglandins $E_1$, $E_2$ and $E_3$. *Immunology* 1995; **76**: 251–257.
105. Willoughby DA, Colville-Nash PR, Seed MP. Inflammation, prostaglandins and loss of function. *J Lipid Mediat Cell Signal* 1993; **6**: 287–293.
106. Huskisson EC, Berry H, Gishen P, Jubb RW, Whitehead J. Effects of antiinflammatory drugs on the progression of osteoarthritis of the knee. *J Rheumatol* 1995; **22**: 1941–1946.
107. Feng L, Xia Y, Garcia GE, Hwang D, Wilson CB. Involvement of reactive oxygen intermediates in cyclooxygenase-2 expression induced by interleukin-1, tumor necrosis factor-α and lipopolysaccharide. *J Clin Invest* 1995; **95**: 1669–1675.
108. Hempel SL, Monick MM, He B, Yano T, Hunninghake GW. Synthesis of prostaglandin H synthase-2 by human alveolar macrophages in response to lipopolysaccharide is inhibited by decreased cell oxidant tone. *J Biol Chem* 1994; **269**: 32979–32984.
109. Shreck R, Bauerle PA. A role for oxygen radicals as second messengers. *Trends in Cell Biology* 1991; **1**: 39–42.
110. Brennan P, O'Neill LAJ. Effects of oxidants and antioxidants on nuclear factor kB activation in three different cell lines: evidence against a universal hypothesis involving oxygen radicals. *Biochim Biophys Acta* 1995; **1260**: 167–175.
111. Davidge ST, Baker PN, McLaughlin MK, Roberts JM. Nitric oxide produced by endothelial cells increases production of eicosanoids through activation of prostaglandin H synthase. *Circ Res* 1995; **77**: 274–283.
112. Salvemini D, Seibert K, Masferrer JL, Misko TP, Currie MG, Needleman P. Endogenous nitric oxide enhances prostaglandin production in a model of renal inflammation. *J Clin Invest* 1994; **93**: 1940–1947.
113. Salvemini D, Settle SL, Masferrer JL, Seibert K, Currie MG, Needleman P. Regulation of prostaglandin production by nitric oxide; an in vivo analysis. *Br J Pharmacol* 1995; **114**: 1171–1178.
114. Sautebin L, Di Rosa M. Nitric oxide modulates prostacyclin biosynthesis in the lung of endotoxin-treated rats. *Eur J Pharmacol* 1994; **262**: 193–196.
115. Sautebin L, Ialenti A, Ianaro A, Di Rosa M. Modulation by nitric oxide of prostaglandin biosynthesis in the rat. *Br J Pharmacol* 1995; **114**: 323–328.
116. Salvemini D, Misko TP, Masferrer JL, Seibert K, Currie MG, Needleman P. Nitric oxide activities cyclooxygenase enzymes. *Proc Natl Acad Sci USA* 1993; **90**: 7240–7244.
117. Tsai A. How does NO activate hemeproteins? *FEBS Lett* 1994; **341**: 141–145.
118. Hajjar DP, Lander HM, Pearce SFA, Upmacis RK, Pomerantz KB. Nitric oxide enhances prostaglandin-h synthase-1 activity by a heme-independent mechanism: evidence implicating nitrosothiols. *J Am Chem Soc* 1995; **117**: 3340–3346.
119. Swierkosz TA, Mitchell JA, Warner TD, Botting RM, Vane JR. Co-induction of nitric oxide synthase and cyclo-oxygenase: interactions between nitric oxide and prostanoids. *Br J Pharmacol* 1995; **114**: 1335–1342.
120. Tetsuka T, Daphna-Iken D, Srivastava SK, Baier LD, DuMaine J, Morrison AR. Cross-talk between cyclooxygenase and nitric oxide pathways: prostaglandin $E_2$ negatively modulates induction of nitric oxide synthase by interleukin 1. *Proc Natl Acad Sci USA* 1994; **91**: 12168–12172.
121. Astin M, Stjernschantz J, Selen G. Role of nitric oxide in $PGF_{2\alpha}$-induced ocular hyperemia. *Exp Eye Res* 1994; **59**: 401–407.
122. Sowa G, Przewlocki R. cAMP analogues and cholera toxin stimulate the accumulation of nitrite in peritoneal macrophage cultures. *Eur J Pharmacol – Mol Pharm Section* 1994; **266**: 125–129.
123. Imai T, Hirata Y, Kanno K, Marumo F. Induction of nitric oxide synthase by cyclic AMP in rat vascular smooth muscle cells. *J Clin Invest* 1994; **93**: 543–549.
124. Hirokawa K, O'Shaughnessy K, Moore K, Ramrakha P, Wilkins MR. Induction of nitric oxide synthase in cultured vascular smooth muscle cells: the role of cyclic AMP. *Br J Pharmacol* 1994; **112**: 396–402.
125. Barker JE, Bakhle YS, Anderson J, Treasure T, Piper PJ. Reciprocal inhibition of nitric oxide and prostacyclin synthesis in human saphenous vein. *Br J Pharmacol* 1996; **118**: 643–648.
126. Angel J, Berenbaum F, Le Denmat C, Nevalainen T, Masliah J, Fournier C. Interleukin-1-induced prostaglandin E2 biosynthesis in human synovial cells involves the activation of cytosolic phospholipase A2 and cyclooxygenase-2. *Eur J Biochem* 1994; **226**: 125–131.
127. Hulkower KI, Wertheimer SJ, Levin W, Coffey JW, Anderson CM, Chen T, DeWitt DL, Crowl RM, Hope WC and Morgan DW. Interleukin-1 beta induces cytosolic phospholipase $A_2$ and prostaglandin H synthase in rheumatoid synovial fibroblasts. Evidence for their roles in the production of prostaglandin $E_2$. *Arth Rheumatism* 1994; **37**: 653–661.
128. Doerfler ME, Weiss J, Clark JD, Elsbach P. Bacterial lipopolysaccharide primes human neutrophils for enhanced release of arachidonic acid and causes phosphorylation of an 85-kD cytosolic phospholipase A2. *J Clin Invest* 1994; **93**: 1583–1591.
129. Forehand JR, Johnston RBJ, Bomalaski JS. Phospholipase A2 activity in human neutrophils. Stimulation by lipopolysaccharide and possible involvement in priming for an enhanced respiratory burst. *J Immunol* 1993; **151**: 4918–4925.
130. Roshak A, Sathe G, Marshall LA. Suppression of monocyte 85-kDa phospholipase A2 by antisense and effects on endotoxin-induced prostaglandin biosynthesis. *J Biol Chem* 1994; **269**: 25999–26005.
131. Jackson BA, Goldstein RH, Roy R, Cozzani M, Taylor L, Polgar P. Effects of transforming growth factor-β and interleukin-1β on expression of cyclooxygenase 1 and 2 and phospholipase $A_2$ mRNA in lung fibroblasts and endothelial cells in culture. *Biochem Biophys Res Commun* 1993; **197**: 1465–1474.
132. Lin LL, Lin AY, DeWitt DL. Interleukin 1α induces the accumulation of cytosolic phospholipase A2 and the release of prostaglandin $E_2$ in human fibroblasts. *J Biol Chem* 1992; **267**: 23451–23454.
133. Chepenik KP, Diaz A, Jimenez SA. Epidermal growth factor coordinately regulates the expression of prostaglandin G/H synthase and cytosolic phospholipase A2 genes in embryonic mouse cells. *J Biol Chem* 1994; **269**: 21786–21792.
134. Shimokawa T, Smith WL. Prostaglandin endoperoxide synthase. The aspirin acetylation region. *J Biol Chem* 1992; **267**: 12387–12392.
135. Flower RJ. Lipocortin and the mechanism of action of the glucocorticoids. *Br J Pharmacol* 1988; **94**: 987–1015.
136. Flower RJ, Rothwell NJ. Lipocortin-1; cellular mechanisms and clinical relevance. *Trends in Pharmacological Sciences* 1994; **15**: 71–76.
137. Blackwell GJ, Flower RJ, Nijkamp FP, Vane JR. Phospholipase $A_2$ activity of guinea-pig isolated perfused lungs: stimulation and inhibition by anti-inflammatory steroids. *Br J Pharmacol* 1978; **62**: 79–89.
138. O'Banion MK, Winn VD, Young DA. cDNA cloning and functional activity of a glucocorticoid-regulated inflammatory cyclooxygenase. *Proc Natl Acad Sci USA* 1992; **89**: 4888–4892.
139. Szczepanski A, Moatter T, Carley WW, Gerritsen ME. Induction of cyclooxygenase II in human synovial microvessel endothelial cells by interleukin-1. Inhibition by glucocorticoids. *Arth Rheumatism* 1994; **37**: 495–503.
140. Yamagata K, Andreasson KI, Kaufman WE, Barnes CA, Worley PF. Expression of a mitogen-inducible cyclooxygenase in brain neurons; regulation by synaptic activity and glucocorticoids. *Neuron* 1993; **11**: 371–386.
141. Kujubu DA, Herschman HR. Dexamethasone inhibits mitogen induction of the TIS10 prostaglandin synthase cyclooxygenase gene. *J Biol Chem* 1992; **267**: 7991–7994.
142. Zakar T, Hirst JJ, Mijovic JE, Olson DM. Glucocorticoids stimulate the expression of prostaglandin endoperoxide H synthase-2 in amnion cells. *Endocrinology* 1995; **136**: 1610–1619.
143. Ishihara O, Matsuoka K, Kinoshita K, Sullivan MH, Elder MG. Interleukin-1 beta-stimulated PGE2 production from early first trimester human decidual cells is inhibited by dexamethasone and progesterone. *Prostaglandins* 1995; **49**: 15–26.
144. Xie W, Fletcher BS, Andersen RD, Herschman HR. v-src induction of the TIS10/PGS2 prostaglandin synthase gene is mediated by an ATF/CRE transcription response element. *Mol Cell Biol* 1994; **14**: 6531–6539.
145. Chanmugam P, Feng L, Liou S, Jang BC, Boudreau M, Yu G, Lee JH, Kwon HJ, Beppu T, Yoshida M and *et al.* Radicicol, a protein tyrosine kinase inhibitor, suppresses the expression of mitogen-inducible cyclooxygenase in macrophages stimulated with lipopolysaccharide and in experimental glomerulonephritis. *J Biol Chem* 1995; **270**: 5418–5426.
146. Shibata Y. Prostaglandin E2 release triggered by phagocytosis of latex particles. A distinct association with prostaglandin synthase isozymes in bone marrow macrophages. *J Immunol* 1995; **154**: 2878–2887.
147. Hamasaki Y, Eling TE. EGF and TPA stimulate de novo synthesis of PGHS-1 and PGHS-2 through different signal transduction pathways. *Prostaglandins Leukotrienes Essential Fatty Acids* 1995; **53**: 225–229.
148. Blanco A, Habib A, Levy-Toledano S, Maclouf J. Involvement of tyrosine kinases in the induction of cyclo-oxygenase-2 in human endothelial cells. *Biochem J* 1995; **312**: 419–423.
149. Stroebel M, Goppelt-Struebe M. Signal transduction pathways responsible for serotonin-mediated prostaglandin G/H synthase expression in rat mesangial cells. *J Biol Chem* 1994; **269**: 22952–22957.
150. Kester M, Coroneos E, Thomas PJ, Dunn MJ. Endothelin stimulates prostaglandin endoperoxide synthase-2 mRNA expression and protein synthesis through a tyrosine kinase-signaling pathway in rat mesangial cells. *J Biol Chem* 1994; **269**: 22574–22580.
151. Rzymkiewicz DM, DuMaine J, Morrison AR. IL-1 beta regulates rat mesangial cyclooxygenase II gene expression by tyrosine phosphorylation. *Kidney International* 1995; **47**: 1354–1363.
152. Marczin N, Papapetropoulos A, Catravas JD. Tyrosine kinase inhibitors suppress endotoxin and IL-1beta-induced NO synthesis in aortic smooth muscle cells. *Am J Physiol* 1993; **265**: H1014–H1018.
153. Kurtz ES, Bailey SC, Arshad F, Lee AA, Przekop PA. Leflunomide: an active anti-inflammatory and anti-proliferative agent in models of dermatologic disease. *Inflamm Res* 1995; **44**: S187–S188.
154. Mattar T, Kochhar K, Bartlett R, Bremer EG, Finnegan A. Inhibition of the epidermal growth factor receptor tyrosine kinase activity by leflunomide. *FEBS Lett* 1993; **334**: 161–164.
155. Xu X, Williams JW, Bremer EG, Finnegan A, Chong AS. Inhibition of protein tyrosine phosphorylation in T cells by a novel immunosuppressive agent, leflunomide. *J Biol Chem* 1995; **270**: 12398–12403.
156. Morris JK, Richards JS. Luteinizing hormone induces prostaglandin endoperoxide synthase-2 and luteinization in vitro by A-kinase and C-kinase pathways. *Endocrinology* 1995; **136**: 1549–1558.
157. Crofford LJ, Wilder RL, Ristimaki AP, Sano H, Remmers EF, Epps HR and Hla T. Cyclooxygenase-1 and -2 expression in rheumatoid synovial tissues. Effects of interleukin-1 beta, phorbol ester, and corticosteroids. *J Clin Invest* 1994; **93**: 1095–1101.
158. Nusing RM, Ullrich V. Regulation of cyclooxygenase and thromboxane synthase in human monocytes. *Eur J Biochem* 1992; **206**: 131–136.
159. Moon CK, Lee SH, Kim JY, Kim MJ, Lee JY, Moon CH. Staurosporine induces de novo synthesis of prostaglandin H synthase-2 in rat alveolar macrophages. *Life Sciences* 1995; **57**: 571–578.
160. Warnock LJ, Hunninghake G. Multiple second messenger pathways regulate IL-1β-induced expression of PGHS-2 mRNA in normal human skin fibroblasts. *J Cell Physiol* 1995; **163**: 172–178.
161. Irvine RF. How is the level of free arachidonic acid controlled in mammalian cells? *Biochem J* 1982; **204**: 3–16.
162. Chilton FH, Fonteh AN, Surette ME, Triggiani M, Winkler JD. Control of arachidonate levels within inflammatory cells. *Biochim Biophys Acta* 1996; **1299**: 1–15.
163. Bakhle YS, Ferreira SH. Lung metabolism of eicosanoids. In: Fishman A, Fisher AB, eds. *Handbook of Physiology.* Bethesda, MD: American Physiological Society, 1985; 365–386.
164. Murakami M, Matsumoto R, Austen KF, Arm JP. Prostaglandin endoperoxide synthase-1 and -2 couple to different transmembrane stimuli to generate prostaglandin $D_2$ in mouse bone marrow-derived mast cells. *J Biol Chem* 1994; **269**: 22269–22275.
165. Mitchell JA, Belvisi MG, Akarasereenont P, Robbins RA, Kwon OJ, Croxtall J, Barnes PJ and Vane JR. Induction of cyclo-oxygenase-2 by cytokines in human pulmonary epithelial cells: regulation by dexamethasone. *Br J Pharmacol* 1994; **113**: 1008–1014.
166. Lee SH, Soyoola E, Chanmugam P, Hart S, Sun W, Zhong H, Liou S, Simmons DL and Hwang D. Selective expression of mitogen-inducible cyclooxygenase in macrophages stimulated with lipopolysaccharide. *J Biol Chem* 1992; **267**: 25934–25938.
167. Karim S, Habib A, Levy-Toledano S, Maclouf J. Cyclooxygenases-1 and -2 of endothelial cells utilize exogenous or endogenous arachidonic acid for transcellular production of thromboxane. *J Biol Chem* 1996; **271**: 12042–12048.
168. Murakami M, Bingham CO, Matsumoto R, Austen KF, Arm JP. IgE-dependent activation of cytokine-primed mouse cultured mast cells induces a delayed phase of prostaglandin $D_2$ generation via prostaglandin endoperoxide synthase-2. *J Immunol* 1995; **155**: 4445–4453.
169. Barnes PJ, Adcock IM. Transcription factors. *Clin Exp Allergy* 1995; **25**: 46–49.
170. Grossman CJ, Wiseman J, Lucas FS, Trevethick MA, Birch PJ. Inhibition of constitutive and inducible cyclooxygenase activity in human platelets and mononuclear cells by NSAIDs and Cox 2 inhibitors. *Inflamm Res* 1995; **44**: 253–257.
171. Klein T, Nusing RM, Pfeilschifter J, Ullrich V. Selective inhibition of cyclooxygenase 2. *Biochem Pharmacol* 1994; **48**: 1605–1610.
172. Laneuville O, Breuer DK, DeWitt DL, Hla T, Funk CD, Smith WL. Differential inhibition of human prostaglandin endoperoxide H synthases-1 and -2 by nonsteroidal anti-inflammatory drugs. *J Pharm Exp Therap* 1994; **271**: 927–934.
173. Battistini B, Botting RM, Bakhle YS. COX-1 and COX-2: toward the development of more selective NSAIDs. *Drug News and Perspectives* 1994; **7**: 501–512.
174. Mitchell JA, Akarasereenont P, Thiemermann C, Flower RJ, Vane JR. Selectivity of non-steroidal anti-inflammatory drugs as inhibitors of constitutive and inducible cyclooxygenase. *Proc Natl Acad Sci USA* 1993; **90**: 11693–11697.
175. Langman MJS, Weil J, Wainwright P, Lawson DH, Rawlins MD, Logan RFA, Murphy M, Vessey, MP and Colin-Jones DG. Risks of bleeding peptic ulcer associated with individual non-steroidal anti-inflammatory drugs. *Lancet* 1994; **343**: 1075–1078.
176. Bennett A, Berti F, Ferreira SH. Nimesulide; a multifactorial therapeutic approach to the inflammatory process?; a 7-year clinical experience. *Drugs* 1993; **46**: 1–283.
177. Wiesenberg-Boettcher I, Schweizer A, Muller K. The pharmacological profile of CGP 28238, a highly potent anti-inflammatory compound. *Agents Actions* 1989; **26**: 240–242.
178. Futaki N, Takahashi S, Yokoyama M, Arai I, Higuchi S, Otomo S. NS-398, a new anti-inflammatory agent, selectively inhibits prostaglandin G/H synthase/cyclooxygenase (COX-2) activity in vitro. *Prostaglandins* 1994; **47**: 55–59.
179. Gans KR, Galbraith W, Roman RJ, Haber SB, Kerr JS, Schmidt WK, Smith C, Hewes WE and Ackerman NR. Anti-inflammatory and safety profile of DuP 697, a novel orally effective prostaglandin synthesis inhibitor. *J Pharm Exp Therap* 1990; **254**: 180–187.
180. Seibert K, Zhang Y, Leahy K, Hauser S, Masferrer J, Perkins W, Lee L and Isakson P. Pharmacological and biochemical demonstration of the role of cyclooxygenase 2 in inflammation and pain. *Proc Natl Acad Sci USA* 1994; **91**: 12013–12017.
181. Chan C-C, Boyce S, Brideau C, Ford-Hutchinson AW, Gordon R, Guay D, Hill RG, Li C-S, Mancini JA, Penneton M, Prasit P, Rasori R, Riendeau D, Roy P, Tagari P, Wong E and Rodger IW. Pharmacology of a selective cyclooxygenase-2 inhibitor, L-745,337: a novel nonsteroidal anti-inflammatory agent with an ulcerogenic sparing effect in rat and non-human primate stomach. *J Pharm Exp Therap* 1995; **274**: 1531–1537.
182. Huff R, Collins P, Kramer S, Seibert K, Koboldt C, Gregory S and Isakson P. A structural feature of N-[2-(cyclohexyloxy)-4-nitrophenyl] methanesulfonamide (NS-398) that governs its selectivity and affinity for cyclooxygenase 2 (COX2). *Inflamm Res* 1995; **44** (suppl 2): S145–S146.
183. Reitz DB, Li JJ, Norton MB, Reinhard EJ, Collins JT, Anderson GD, Gregory SA, Koboldt CM, Perkins WE, Seibert K and Isakson PC. Selective cyclooxygenase inhibitors: novel 1,2-diarylcyclopentenes are potent and orally active COX-2 inhibitors. *J Med Chem* 1994; **37**: 3878–3881.
184. Leblanc Y, Gauthier J, Ethier D, Guay J, Mancini JA, Riendeau D, Tagari P, Vickers PJ, Wong E and Prasit P. Synthesis and biological evaluation of 2,3-diarylthiophenes as selective Cox-2 and Cox-1 inhibitors. *Bioorg Med Chem Lett* 1995; **5**: 2123–2128.
185. Tanaka K, Kawasaki H, Kurata K, Aikawa Y, Tsukamoto Y, Inaba T. T-614, a novel anti-rheumatic drug, inhibits both the activity and induction of cyclooxygenase-2 (COX-2) in cultured fibroblasts. *Jpn J Pharmacol* 1995; **67**: 305–314.
186. Eberhart CE, Dubois RN. Eicosanoids and the gastrointestinal tract. *Gastroenterology* 1995; **109**: 285–301.
187. Giovanucci E, Egan KM, Hunter DJ, Stampfer MJ, Colditz GA, Willett WC and Speizer FE. Aspirin and the risk of colorectal cancer in women. *New Engl J Med* 1995; **333**: 609–614.
188. Muller-Decker K, Scholz K, Marks F, Furstenberger G. Differential expression of prostaglandin H synthase isozymes during multistage carcinogenesis in mouse epidermis. *Mol Carcinog* 1995; **12**: 31–41.
189. Kargman SL, O'Neill GP, Vickers PJ, Evans JF, Mancini JA, Jothy S. Expression of prostaglandin G/H synthase-1 and -2 protein in human colon cancer. *Cancer Res* 1995; **55**: 2556–2559.
190. Lu X, Xie W, Reed D, Bradshaw WS, Simmons DL. Nonsteroidal antiinflammatory drugs cause apoptosis and induce cyclooxygenases in chicken embryo fibroblasts. *Proc Natl Acad Sci USA* 1995; **92**: 7961–7965.
191. Hempel SL, Monick MM, Hunninghake GW. Lipopolysaccharide induces prostaglandin H synthase-2 protein and mRNA in human alveolar macrophages and blood monocytes. *J Clin Invest* 1994; **93**: 391–396.
192. Gu W, Rice GE, Brennecke SP. Effects of lipopolysaccharide on human placental prostaglandin F2 alpha production in vitro. *Prostaglandins Leukotrienes Essential Fatty Acids* 1994; **50**: 311–315.
193. Arias-Negrete S, Keller K, Chadee K. Proinflammatory cytokines regulate cyclooxygenase-2 mRNA expression in human macrophages. *Biochem Biophys Res Commun* 1995; **208**: 582–589.
194. Topley N, Petersen MM, Mackenzie R, Neubauer A, Stylianou E, Kaever V, Davies M, Coles GA, Jorres A and Williams JD. Human peritoneal mesothelial cell prostaglandin synthesis: induction of cyclooxygenase mRNA by peritoneal macrophage-derived cytokines. *Kidney Int* 1994; **46**: 900–909.
195. Knott I, Dieu M, Burton M, Houbion A, Remacle J, Raes M. Induction of cyclooxygenase by interleukin 1: comparative study between human synovial cells and chondrocytes. *J Rheumatol* 1994; **21**: 462–466.
196. Endo T, Ogushi F, Sone S, Ogura T, Taketani Y, Hayashi Y, Ueda N and Yamamoto S. Induction of cyclooxygenase-2 is responsible for interleukin-1 beta-dependent prostaglandin E2 synthesis by human lung fibroblasts. *Am J Respir Cell Mol Biol* 1995; **12**: 358–365.
197. Claria J, Serhan CN. Aspirin triggers previously undescribed bioactive eicosanoids by human endothelial cell-leukocyte interactions. *Proc Natl Acad Sci USA* 1995; **92**: 9475–9479.
198. Lonchampt MO, Schulz J, Mabille K, Chabrier PE, Braquet P. Interleukin-1 activates preferentially cyclooxygenase rather than NO synthase pathway in human smooth muscle cells. *Agents Actions* 1994; **41** (special conference issue): C164–C165.
199. Kennard EA, Zimmerman PD, Friedman CI, Kniss DA. Interleukin-1β induces cyclooxygenase-2 in cultured human decidual cells. *Am J Reprod Immunol* 1995; **34**: 65–71.
200. Yang CY, Meng CL. Regulation of PG synthase by EGF and PDGF in human oral, breast, stomach, and fibrosarcoma cancer cell lines. *J Dent Res* 1994; **73**: 1407–1415.
201. Kelner MJ, Uglik SF. Mechanism of prostaglandin $E_2$ release and increase in $PGH_2/PGE_2$ isomerase activity by PDGF: involvement of nitric oxide. *Arch Biochem Biophys* 1994; **312**: 240–243.
202. Hoff T, Kaever V, Resch K, DeWitt DL, Goppelt-Struebe M. Prostaglandin endoperoxide synthase-1 and synthase-2 expression in differentiating human monocytic cells. *Agents Actions* 1994; **41** (special conference issue): C159–C161.
203. Zakar T, Teixeira FJ, Hirst JJ, Guo F, MacLeod EA, Olson DM. Regulation of prostaglandin endoperoxide H synthase by glucocorticoids and activators of protein kinase C in the human amnion. *J Reprod Fertil* 1994; **100**: 43–50.
204. Matijevic-Aleksic N, Sanduja SK, Wang LH, Wu KK. Differential expression of thromboxane A synthase and prostaglandin H synthase in megakaryocytic cell line. *Biochim Biophys Acta* 1995; **1269**: 167–175.
205. Slater D, Berger L, Newton R, Moore G, Bennett P. The relative abundance of type 1 to type 2 cyclo-oxygenase mRNA in human amnion at term. *Biochem Biophys Res Commun* 1994; **198**: 304–308.
206. Johnson RD, Walsh SW, Everson WV, Nelson DM. Differentiation and growth on a fibrin matrix modulate the cyclooxygenase expression and thromboxane production by cultured human placental trophoblasts. *Prostaglandins Leukotrienes Essential Fatty Acids* 1995; **52**: 21–27.
207. Hirst JJ, Teixeira FJ, Zakar T, Olson DM. Prostaglandin endoperoxide-H synthase-1 and -2 messenger ribonucleic acid levels in human amnion with spontaneous labor onset. *J Clin Endocrinol Metab* 1995; **80**: 517–523.
208. Slater DM, Berger LC, Newton R, Moore GE, Bennett PR. Expression of cyclooxygenase types 1 and 2 in human fetal membranes at term. *Am J Obstet Gynecol* 1995; **172**: 77–82.
209. Zuo J, Lei ZM, Rao CV, Pietrantoni M, Cook VD. Differential cyclooxygenase-1 and -2 gene expression in human myometria from preterm and term deliveries. *J Clin Endocrinol Metab* 1994; **79**: 894–899.
210. Teixeira FJ, Zakar T, Hirst JJ, Guo F, Sadowsky DW, Machin G, Demianczuk N, Resch B and Olson DM. Prostaglandin endoperoxide-H synthase (PGHS) activity and immunoreactive PGHS-1 and PGHS-2 levels in human amnion throughout gestation, at term, and during labor. *J Clin Endocrinol Metab* 1994; **78**: 1396–1402.
211. Divers MJ, Lilford RJ, Miller D, Bulmer JN. Cyclo-oxygenase distribution in human placenta and decidua does not change with labour after term or preterm delivery. *Gynecol Obstet Invest* 1995; **39**: 157–161.
212. Freed KA, Aitken MA, Brennecke SP, Rice GE. Prostaglandin G/H synthase-1 messenger RNA relative abundance in human amnion, choriodecidua and placenta before, during and after spontaneous-onset labour at term. *Gynecol Obstet Invest* 1995; **39**: 73–78.
213. Warner TD, Amirmansour C, Vane JR. Nimesulide: selectivity for cyclo-oxygenase 2 over cyclo-oxygenase 1. *Inflamm Res* 1995; **44**: 274S.
214. Bottcher I, Schweizer A, Glatt M, Werner H. A sulphonamido-indanone CGP 28237 (ZK 34228), a novel non-steroidal anti-inflammatory agent without gastrointestinal ulcerogenicity in rats. *Drugs Under Experimental and Clinical Research* 1987; **13**: 237–245.
215. Futaki N, Yoshikawa K, Hamasaka Y, Arai I, Higuchi S, Iizuka H and Otomo S. NS-398, a novel non-steroidal anti-inflammatory drug with potent analgesic and antipyretic effects which causes minimal stomach lesions. *General Pharmacology* 1993; **24**: 105–110.
216. Engelhardt G, Bogel R, Schnitzler C, Utzmann R. Meloxicam: influence on arachidonic acid metabolism; *in vitro* findings. *Biochem Pharmacol* 1995; **51**: 21–28.
217. Engelhardt G, Homma D, Schlegel K, Utzmann R, Schnitzler C. Anti-inflammatory, analgesic, antipyretic and related properties of meloxicam, a new non-steroidal anti-inflammatory agent with favourable gastrointestinal tolerance. *Inflamm Res* 1995; **44**: 423–433.

ACKNOWLEDGEMENTS. The William Harvey Research Institute is supported by grants from the ONO Pharmaceutical Company, Schwarz Pharma Ltd, and the Servier International Research Institute.

**Received 5 July 1996; accepted 16 July 1996**
